# Ozone ultrafine bubble water induces the cellular signaling involved in oxidative stress responses in human periodontal ligament fibroblasts

**DOI:** 10.1080/14686996.2019.1614980

**Published:** 2019-06-13

**Authors:** Anongwee Leewananthawet, Shinichi Arakawa, Tokuju Okano, Ryo Daitoku Kinoshita, Hiroshi Ashida, Yuichi Izumi, Toshihiko Suzuki

**Affiliations:** a Department of Bacterial Pathogenesis, Infection and Host Response, Graduate School of Medical and Dental Sciences, Tokyo Medical and Dental University, Tokyo, Japan; b Department of Periodontology, Graduate School of Medical and Dental Sciences, Tokyo Medical and Dental University, Tokyo, Japan; c Department of Lifetime Oral Health Care Science, Graduate School of Medical and Dental Sciences, Tokyo Medical and Dental University, Tokyo, Japan; d Oral Care and Perio Center, Southern TOHOKU Research Institute for Neuroscience, Fukushima, Japan

**Keywords:** Ozone ultrafine bubble water (OUFBW), aqueous ozone, cellular response, periodontitis, 30 Bio-inspired and biomedical materials, 211 Scaffold / Tissue engineering / Drug delivery

## Abstract

Periodontitis is a chronic inflammatory disease caused by oral microorganisms in the subgingival biofilm. Stable aqueous ozone ultrafine bubble water (OUFBW) has recently begun to be used as an antiseptic in the treatment of periodontitis. The effectiveness of OUFBW is thought to depend on the bactericidal actions of dissolved ozone exerted via its oxidizing effect. On the other hand, the effects of ozone on the periodontal tissues are largely unknown. In this paper we examined the cellular responses after OUFBW treatment. Human primary periodontal ligament fibroblasts (hPDLFs) or Ca9-22 human gingival epithelial cells were treated with OUFBW or UV-inactivated OUFBW. The production of reactive oxygen species (ROS), the activation of mitogen-activated protein kinase (MAPK) and the nuclear factor-kappa B (NF-κB) activation were analyzed. The transcript profiles of hPDLFs after OUFBW treatment were also analyzed by RNA sequencing (RNA-seq). Our results showed that OUFBW induces oxidative stress by generating ROS, which, in turn, activated the MAPK pathway. OUFBW triggered activation of c-Fos, a major component of the transcription factor activator protein 1 (AP-1), and also nuclear factor erythroid 2 (NF-E2)-related factor 2 (Nrf2), which possessed a high sensitivity to oxidative stress. The results of RNA-seq analysis revealed that the numerous genes involved in oxidative stress responses or MAPK signaling pathway were up-regulated after OUFBW treatment. Investigation of the signaling pathways activated by OUFBW highlights another aspect of the biological roles of OUFBW, in addition to its bactericidal activity, in the treatment of periodontitis.

## GRAPHICAL ABSTRACT

**Figure:**
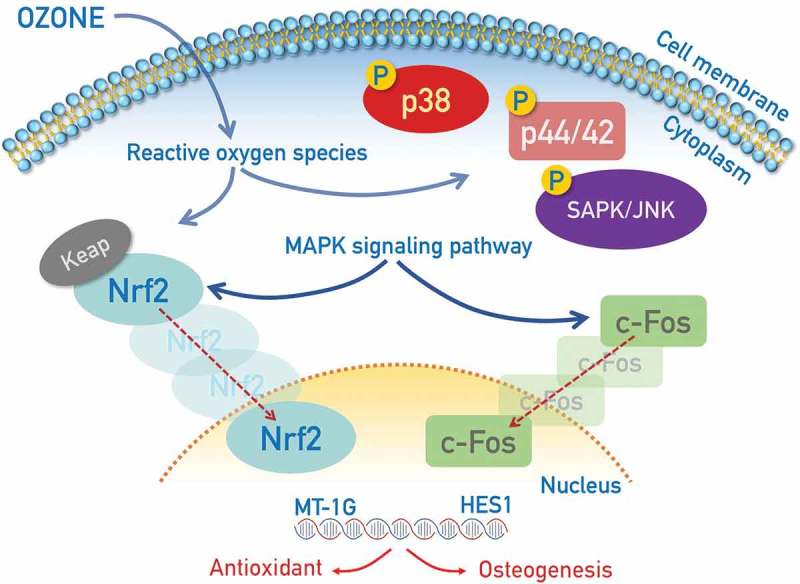

## 1. Introduction

Ozone (O_3_) is a strong oxidizing agent that is widely used as an antiseptic in the food industry and dental fields. Regarding practical application of its antimicrobial activity, ozone has been used for water purification and food preservation, and more recently, as a disinfectant in the treatment of dental caries, endodontic lesions and periodontal diseases [1–7]. The strong bactericidal efficacy of ozone results from its strong oxidizing activity. Meanwhile, ozone is also known to induce oxidative stress in cells via stimulating the production of reactive oxygen species (ROS). Note that inhalation of high concentrations of gaseous ozone has been suggested as a possible risk factor in the development of asthma and allergic airway inflammation [8,9].

Aqueous ozone has been widely used in clinical practice, however, it is unstable. Ozonated water has a half-life about 20–30 min, after which ozone converts into oxygen. To overcome this instability of ozonated water, ozone ultrafine bubble water (OUFBW) has been developed by collapsing micro-bubbles of ozone in an electrolyte solution with a physical stimulus [10]. The oxidizing ability of ozone remains stable for more than 6 months in an electrolyte solution stored under a protected condition against ultraviolet (UV) rays. A previous randomized controlled trial demonstrated that subgingival irrigation with OUFBW improved the periodontal status in patients suffering from periodontitis [11]. According to another study, the effect of OUFBW is attributable to its bactericidal activity, presumably exerted via a free-radical-mediated oxidation reaction, and OUFBW had no impact on the tissue viability in a reconstituted oral tissue model *in vitro* [12]. On the other hand, there are no reports regarding the potential ability of OUFBW to stimulate regeneration of the lost supporting periodontal tissues in periodontitis.

In this study, we demonstrated that OUFBW induced oxidative stress in cells, mediated by the production of ROS; in turn, the oxidative stress induced activation of the mitogen-activated protein kinase (MAPK) pathway in the cells. OUFBW triggered the activation of c-Fos, a major component of the transcription factor activator protein 1 (AP-1), and also nuclear factor erythroid 2 (NF-E2)-related factor 2 (Nrf2), a transcription factor with a high sensitivity to oxidative stress. Using RNA sequencing (RNA-seq) analysis, it was revealed that the numerous genes involved in oxidative stress responses or MAPK signaling pathway were actually up-regulated after OUFBW treatment.

This investigation of the signaling pathways activated by OUFBW serves to highlight other biological roles of OUFBW, in addition to its bactericidal activity, in the treatment of periodontitis.

## 2. Material and methods

### 2.1. Ozone ultrafine bubble water

Commercially available OUFBW (Kyocera Corp., Japan) was used for the experiments in this study. The concentration of ozone in the OUFBW was 2.5 ppm, as measured with an ozone meter (AOM-05, Sato Shoji Inc., Japan) before each experiment. The particle concentration of OUFBW was 1.68 × 10^9^ particles/mL, as determined using the nanoparticle multi-analyzer: qNano (Meiwafosis Co., Ltd, Japan). Inactivation of ozone was performed by UV irradiation (15 W UV fluorescent lamp, GL15, TOSHIBA) from a distance of 65 cm for 4 h, and absence of ozone in the water was confirmed.

### 2.2. Cell culture

Human primary periodontal ligament fibroblasts (hPDLFs) isolated from a 16-year-old male were purchased from Lonza Walkersville, Inc., USA (CC-7049, Clonetics Human Periodontal Ligament Fibroblast Cell Systems) and maintained in phenol red-free fibroblast basal medium (C-23215, PromoCell, GmbH, Germany) with growth supplements kit (CC-4181, SCGM SingleQuots, Lonza Walkersville, Inc., USA), containing insulin, fetal bovine serum (FBS), gentamicin sulfate/amphotericin-B (GA-1000) and human fibroblast growth factor-basic (rhFGF-B). The final concentration of growth supplements was prepared according to the manufacturer’s instructions. The Ca9-22 human oral epithelial cell line was maintained in Eagle’s minimal essential medium (MEM) and 10% FBS was also used.

### 2.3. Cell viability assessment

The viability of cells was evaluated by the MTT[3-(4,5–dimethythiazol–2-yl)-2,5-diphenyl tetrazolium bromide] (Sigma-Aldrich) assay. In brief, hPDLFs and Ca9-22 suspension (0.1 mL/well) were seeded onto 96‑well plates at a cell density of 7 × 10^3^ and 37.5 × 10^3^ cells/well, respectively, 24 h prior to treatment. Then, each well was washed with 0.2 mL of phosphate-buffered saline (PBS). The cells were then left untreated (control), treated with inactive OUFBW as the negative control, or treated with OUFBW (0.1 mL/well) for 1 and 10 min, followed by incubation in the medium for 3 h at 37°C in an atmosphere containing 5% CO_2_. For the assessment, 0.01 mL of MTT labeling reagent (0.5 mg/mL) was added to each well. After incubation at 37°C under 5% CO_2_ for 4 h, 0.1 mL of solubilization solution was added to each well, followed by incubation overnight at 37°C in an atmosphere containing 5% CO_2_. The purple formazan crystals formed were measured at 595 nm using a microplate reader (Bio-Rad). The intensity of the color produced was directly proportional to the number of living cells. The cell viability was determined as the averaged absorbance.

### 2.4. Detection of intracellular ROS

Intracellular ROS generation was determined by staining with CellROX Oxidative Stress Reagents (CellROX Deep Red Reagent #C10422, Life Technologies), in accordance with the manufacturer’s instructions. Briefly, the cells were plated in 24-well plates at a cell density of 0.9 × 10^5^ cells/well, 24 h prior to treatment. Each well was washed with PBS, and then 1 mL/well of basal medium or inactive OUFBW was added in the negative control groups and OUFBW in the experimental group. H_2_O_2_ (100 µM) was used as the positive control for intracellular ROS accumulation. After incubation for 30 min at 37°C in the presence of 5% CO_2_, all the stimulants were replaced by basal medium, followed by further incubation for 30 min. For the detection of oxidative stress, the cells were stained with CellROX Deep Red Reagent at a final concentration of 5 μM for 30 min at 37°C, followed by three washes with PBS. Subsequently, the cells were fixed with 4% paraformaldehyde for 15 min at room temperature and washed three times with Tris-buffered saline (TBS). Nuclei were counterstained with 4ʹ,6-diamidino-2-phenylindole (DAPI, 1:2500) for 30 min in the dark at room temperature. After incubation, the cells were washed three times with TBS containing 0.05% Tween-20. The ROS production was immediately imaged by inverted digital fluorescence microscopy (DMI 6000B, Leica).

### 2.5. Western blot analysis

The cells were seeded at a density of 0.75 × 10^6^ cells per well in six-well plates and incubated 24 h prior to treatment. The wells were treated with OUFBW and inactive OUFBW for 10 min and exchanged with the medium, following incubation for the indicated times. As the positive controls, the cells were treated with 500 ng/mL of tumor necrosis factor alpha (TNF-α, Peprotech) for 2 h or UV irradiation for 20 min, following incubation for 30 min. After stimulation, the cells were lysed with lysis buffer (25 mM Tris-HCl, pH 7.4, 150 mM NaCl, 1% NP-40, complete protease inhibitor cocktail [Roche Diagnostics]). The samples were subjected to Western blot analysis using the following antibodies: phospho-MAPK family antibody sampler kit (#9910, Cell Signaling), MAPK family antibody sampler kit (#9926, Cell Signaling), phospho-IκBα mouse antibody (#2859, Cell Signaling), IκBα mouse antibody (#4814, Cell Signaling), and actin mouse antibody, clone 4 (#MAB1501, Merck).

### 2.6. Immunofluorescence

hPDLFs cells were cultured on glass coverslips at a cell density of 0.18 × 10^6^ cells/well in six‑well plates. After 24 h, the cells were treated with OUFBW and inactive OUFBW for 10 min. The stimulants were then replaced by the medium, followed by incubation at 37°C. For staining of c-Fos or Nrf2, OUFBW-treated cells were incubated for 30 min and 6 h, respectively. As a positive control for activation of c-Fos, the cells were treated with 1 mM phorbol 12-myristate 13-acetate (PMA, Sigma) for 30 min. The cells were also treated with 500 ng/mL of TNF-⍺ for 4 h as a control for Nrf2 activation. The treated cells were fixed with 4% paraformaldehyde in phosphate buffer solution for 10 min at room temperature and then immunostained using antibodies c-Fos antibody (GTX129846, GeneTex) or anti-Nrf2 Rabbit antibody (#12721, Cell Signaling), followed by the secondary antibody fluorescein isothiocyanate (FITC)-labeled anti-rabbit donkey IgG (711–095-152, Jackson ImmunoResearch) and DAPI. The samples were analyzed by inverted digital fluorescence microscopy.

### 2.7. Preparation of RNA

For RNA-seq analysis, the cells were seeded onto six-well plates at a density of 0.4 × 10^6^ cells/well and incubated 24 h prior to treatment. After washing with PBS once, the cells were treated with inactive OUFBW or OUFBW for 10 min, then incubated in basal medium for 3 h. Total RNA was isolated from hPDLFs by using the Direct-zol RNA Miniprep Plus (Zymo research; R2070, USA) according to the manufacturer’s instructions. Total RNA of each sample was quantified and qualified by Agilent 2100 Bioanalyzer (Agilent Technologies, CA, USA), NanoDrop (Thermo Fisher Scientific Inc.) and 1.5% agarose gel. The sample libraries were constructed according to the manufacturer’s protocol (NEBNext UltraTM RNA Library Prep Kit for Illumina).

### 2.8. RNA sequencing (RNA-seq) analysis

Sequencing was carried out using a 2 × 150 bp paired-end (PE) configuration; image analysis and base calling were conducted by the HiSeq Control Software (HCS) + OLB + GAPipeline-1.6 (Illumina) on the HiSeq instrument. In order to remove technical sequences including adapters, polymerase chain reaction (PCR) primers, or fragments thereof, bases lower than 20 bp were processed by Trimmomatic (v0.30) to be high-quality clean data. The clean data were aligned to Hg19 (v_hg19) reference genome via software Hisat2 (v2.0.1). Differential expression analysis was performed with the Bioconductor package edgeR (v3.4.6). DESeq (v1.6.3), a model based on the negative binomial distribution, was used for samples with biological replicates. The gene expression levels were calculated by using fragments per kilobase per million reads (FPKM) based on reading counts from HT-seq (v0.6.1) [13]. After adjusted by Benjamini and Hochberg’s approach for controlling the false discovery rate, the *p*-value of genes <0.05 and the absolute value of fold change ≥2 were adopted to detect differentially expressed genes. GO-Term Finder was used to identify Gene Ontology (GO) terms that annotate a list of enriched genes with a significant *p*-value < 0.05. Kyoto Encyclopedia of Genes and Genomes (KEGG) is a collection of databases dealing with genomes, biological pathways, diseases, drugs, and chemical substances. We used custom scripts to enrich the significant differential expression gene in KEGG pathways.

### 2.9. Statistical analysis

All data are presented as the means ± standard deviation of at least three determinations per experimental condition. All experiments were performed at least three times, and representative results are shown in the figures. Statistical analyses were performed using unpaired two-tailed Student’s *t*-tests. Differences at *p*-values of <0.05 were considered as being significant.

## 3. Results

### 3.1. OUFBW has no significant cytotoxic effects at the cellular level

A previous study showed that OUFBW did not exhibit any significant cytotoxicity against oral tissues in a human reconstituted 3D tissue model [12]. To investigate the cytotoxicity of OUFBW at the cellular level, we used human primary periodontal ligament fibroblasts (hPDLFs), since these cells play an important role in the maintenance and regeneration of periodontal tissues. The cells were exposed to OUFBW for 1 or 10 min, because the treatment time with OUFBW is assumed to be less than 10 min in clinical use, and then the cells were further incubated for 3 h in the medium. For rigorous control of the OUFBW, the water was exposed to UV irradiation for 4 h to completely inactivate the ozone in the solution. The concentration of ozone was quantified, and it was confirmed that the water did not contain any ozone.

**Figure 1.**
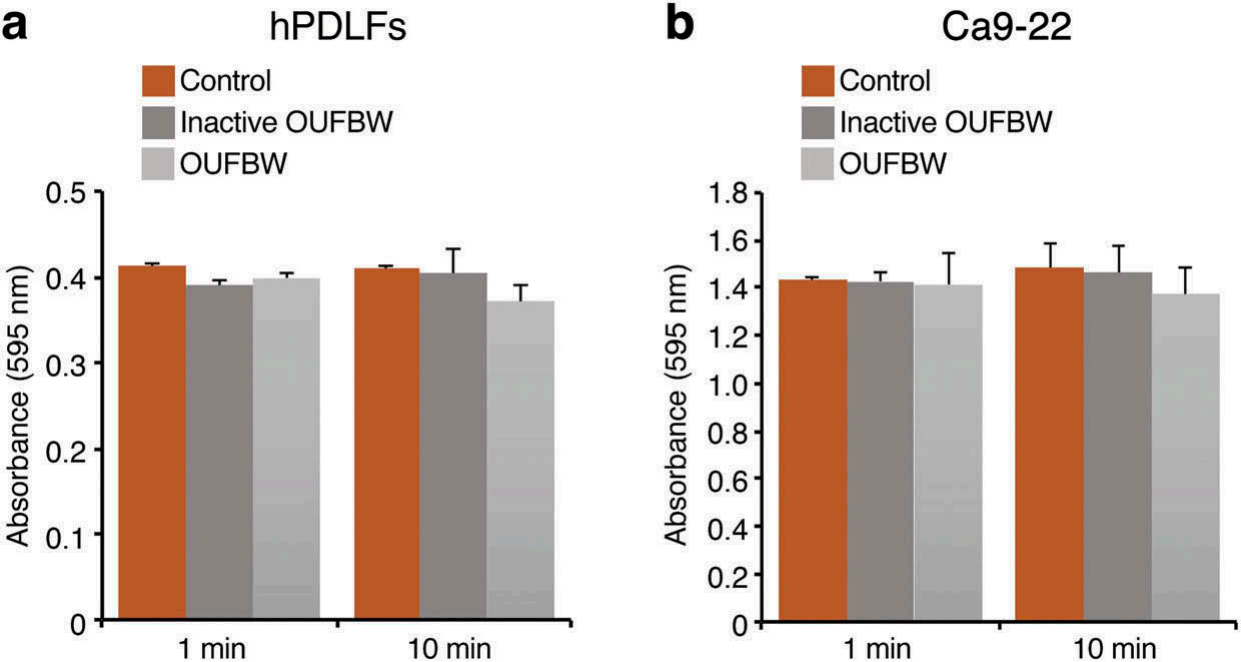
Cell viability after 1 and 10 min of exposure to the medium or inactive OUFBW (negative control group), and OUFBW. hPDLFs (a) and Ca9-22 cells (b) were exposed to OUFBW for 1 or 10 min, followed by further incubation for 3 h in the medium. The cells treated with medium alone were used only as the negative control. There were no significant differences in the cell viability between the OUFBW-stimulated groups and negative control groups (n = 4). Data are shown as means ± SD.

The cell viability of hPDLFs was examined by the MTT assay. As shown in Figure 1(a), OUFBW had no significant cytotoxic effect against the hPDLFs during the observation period, just like inactive OUFBW and negative control. We also examined the cell viability of the Ca9-22 human oral epithelial cell line and observed no cytotoxicity (Figure 1(b)). Furthermore, approximately 80% of cells were still surviving when the OUFBW exposure time was prolonged to 30 min and the incubation time was extended to 24 or 48 h (data not shown).

### 3.2. OUFBW stimulates the production of reactive oxygen species in the treated cells

**Figure 2.**
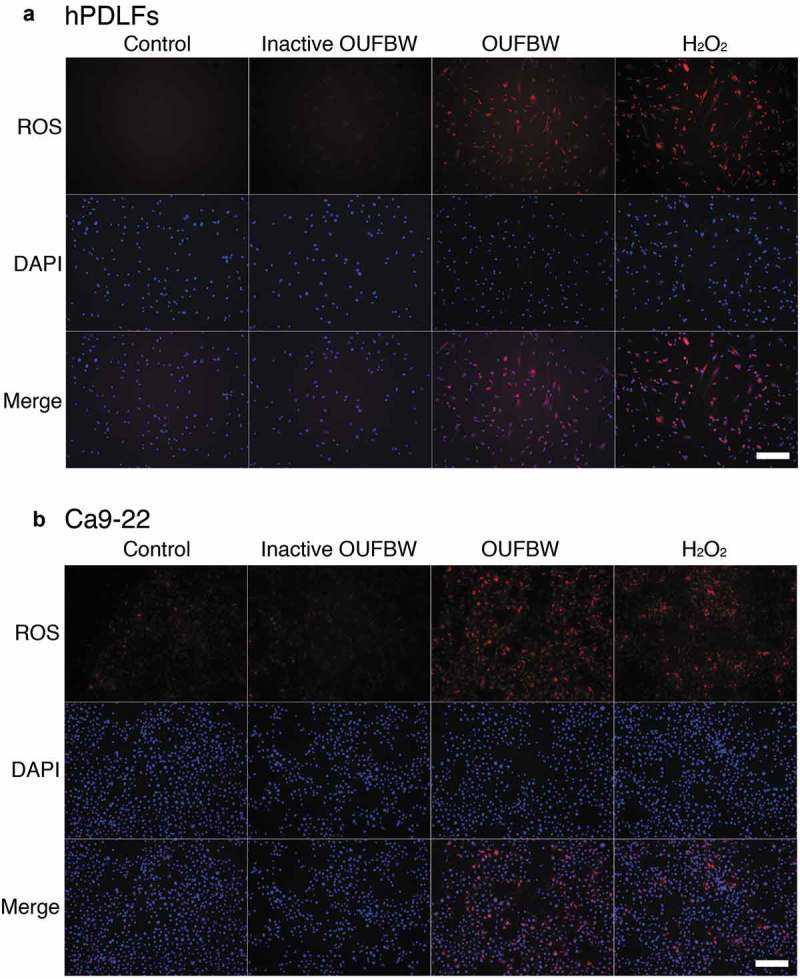
Production of ROS induced by OUFBW treatment in the hPDLFs and Ca9-22 cells. The cells were treated for 30 min with the medium (control), inactive OUFBW or OUFBW or 100 µM of H_2_O_2_ (positive control). Following stimulation, the cells were incubated with the medium for 30 min. The cells were then stained with 5 µM CellROX deep red reagent and DAPI, and then analyzed by fluorescence microscopy. Scale bars = 200 µm.

Ozone is one of the known inducer of oxidative stress. This is achieved by elevating the level of ROS through the biological activity within the cells. ROS have been implicated in various important cellular processes, including cell signaling, homeostasis and induction of cell death. Therefore, we examined whether ROS are also produced in OUFBW-exposed hPDLFs, using a fluorescent probe. As shown in Figure 2(a), strong fluorescence was observed in the OUFBW-treated hPDLFs, but not in the cells treated with inactive OUFBW, suggesting that induction of intracellular ROS depends on the ozone in OUFBW, mediated by oxidative stress. Similar results were obtained in the experiments conducted using Ca9-22 human gingival epithelial cells (Figure 2(b)).

### 3.3. The cellular p38 mitogen-activated protein kinase (p38 MAPK) pathway is activated by ozone exposure

**Figure 3.**
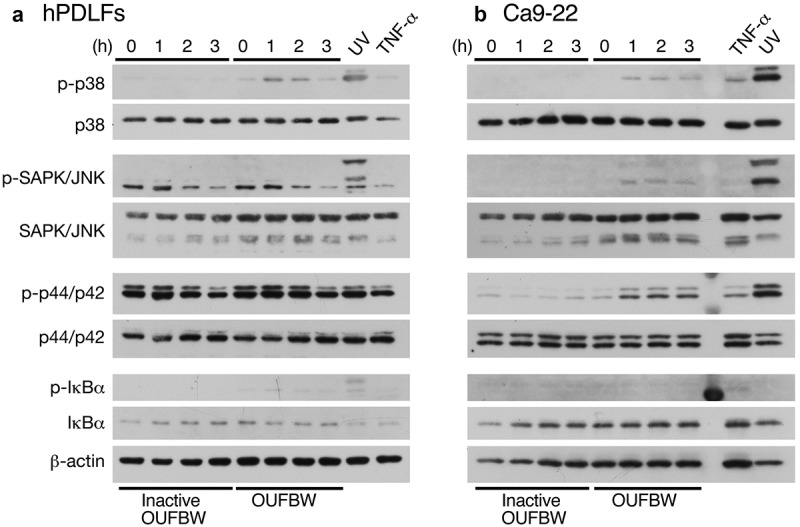
Phosphorylation of MAPK induced by OUFBW. The hPDLFs (a) and Ca9-22 cells (b) were stimulated with medium, inactive OUFBW or OUFBW for 10 min. Cells stimulated with ultraviolet irradiation for 20 min or 500 ng/mL of TNF-α for 2 h were used as the positive controls. Activation of the MAPK cascade was analyzed to detect the phosphorylation of p38 MAPK, SAPK/JNK, or p44/p42 MAPK (Erk1/2) using their phosphorylation-specific antibodies. The levels of β-actin as the loading control were also examined.

For evaluating the cell signaling induced by OUFBW, we firstly analyzed the MAPK cascade and the NF-κB activation pathway in hPDLFs by Western blot analysis using phosphorylation-specific antibodies. The strong oxidative stress induced by compounds such as hydrogen peroxide (H_2_O_2_) triggers activation of the MAPK pathways. In fact, the activation of p38 MAPK, stress-activated protein kinase (SAPK)/c-Jun N-terminal kinase (JNK), or p44/p42 MAPK (Erk1/2), has been reported in airway inflammation [14,15]. The phosphorylation state of Erk1/2 was apparently constitutively activated in hPDLFs (Figure 3(a)). This could be because the rhFGF-B or insulin was added to the medium for maintaining cell survival and proliferation. However, phosphorylation of p38 MAPK was clearly induced after treatment with OUFBW, but not in those exposed to UV-inactivated OUFBW, indicating that the p38 MAPK activation was ozone-dependent (Figure 3(a)). On the other hand, no phosphorylation of either SAPK/JNK or IκBα was detected after OUFBW treatment, suggesting that SAPK/JNK or NF-κB-mediated signaling is not involved in ozone-triggered signaling. Next, we conducted similar experiments using Ca9-22 cells. As shown in Figure 3(b), similar to the case in the hPDLFs, UV irradiation induced activation of all MAPK cascades in the Ca9-22 cells, and less activated after TNF-α treatment in Ca9-22 cells. In contrast to the case in the hPDLFs, the OUFBW treatment of Ca9-22 cells induced the phosphorylation of p38, SAPK/JNK and p44/p42 MAPK, but not that of IκBα.

The different degrees of phosphorylation of MAPK could be due to the different physiological states between the primary cells and the established cancer cell line. Therefore, we conducted the further analyses using the primary hPDLFs.

### 3.4. The nuclear translocation of c-Fos and Nrf2 induced by OUFBW

**Figure 4.**
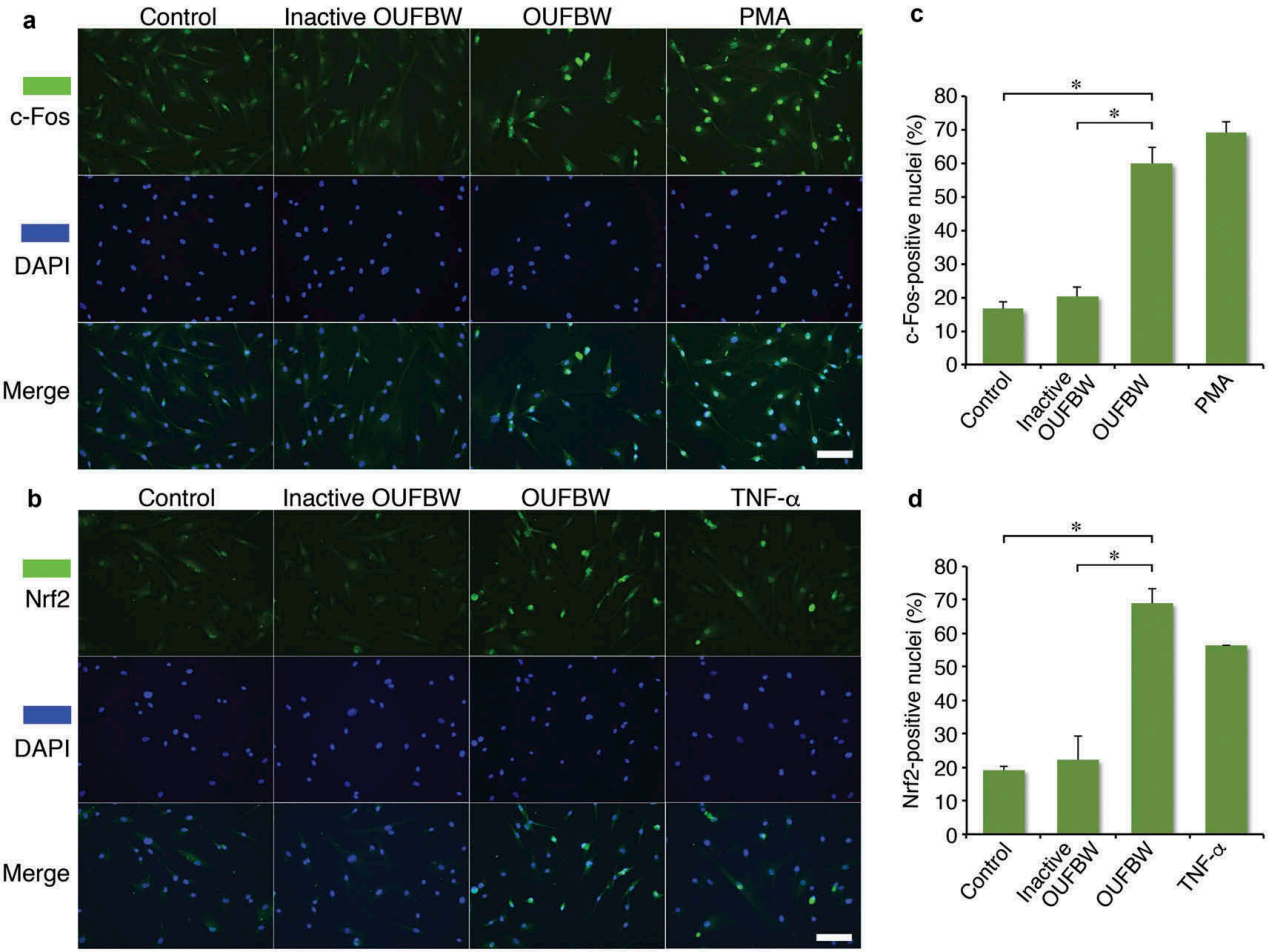
Nuclear translocation of c-Fos and Nrf2 induced by OUFBW. The hPDLFs were stimulated with the medium, inactive OUFBW or OUFBW for 10 min. After the stimulation, the cells were incubated with the medium for 30 min or 6 h for evaluating the localization of c-Fos or Nrf2, respectively. Cells stimulated with 1 mM PMA for 30 min or 500 ng/mL TNF-α for 4 h were used as the positive controls. Cells were then fixed and immunostained with FITC-anti-c-Fos (a) or FITC-anti-Nrf2 (b). The nuclei were stained with DAPI (blue). Scale bars = 100 µm. (c and d) Percentage of the cells showing nuclear translocation of c-Fos (c) or Nrf2 (d). *p < 0.01 (Student’s *t*-test). Data are shown as means ± SD.

AP-1, a family of dimeric transcription factors, including c-Jun and c-Fos, activates the transcription of downstream gene expressions by translocating from the cytoplasm to the nuclei upon stimulation by oxidative stress. Since the p38 MAPK signaling cascade is involved in AP-1 activation by H_2_O_2_ [14], we examined the localization of c-Fos after treatment with OUFBW. The strong fluorescence signal of c-Fos in the nuclei was detected in the cells treated with OUFBW as well as PMA, but not in those exposed to inactive OUFBW (Figure 4(a)). Quantified analysis of the nuclear localization of c-Fos supported the results (Figure 4(c)), suggesting the activation of c-Fos by the ozone in OUFBW. The Nrf2-mediated pathway is the major regulator of cytoprotective responses to oxidative stress [16]. Also, activation of MAPK pathways induces antioxidant response element (ARE)-mediated gene expression via an Nrf2-dependent mechanism [17]. Oxidative stress stabilizes Nrf2 by preventing association with inhibitor protein Keap1, followed by translocation of Nrf2 into the nuclei [16]. Therefore, we next examined the cellular location of Nrf2 after treatment with OUFBW. Similar to the case of c-Fos, Nrf2 was translocated into the nuclei of the cells treated with OUFBW (Figure 4(b,d)). Taken together, these data suggest that the ozone in OUFBW stimulates cell signaling via c-Fos or Nrf2 in the hPDLFs.

### 3.5. Changes in transcript profiles of hPDLFs after OUFBW treatment

**Figure 5.**
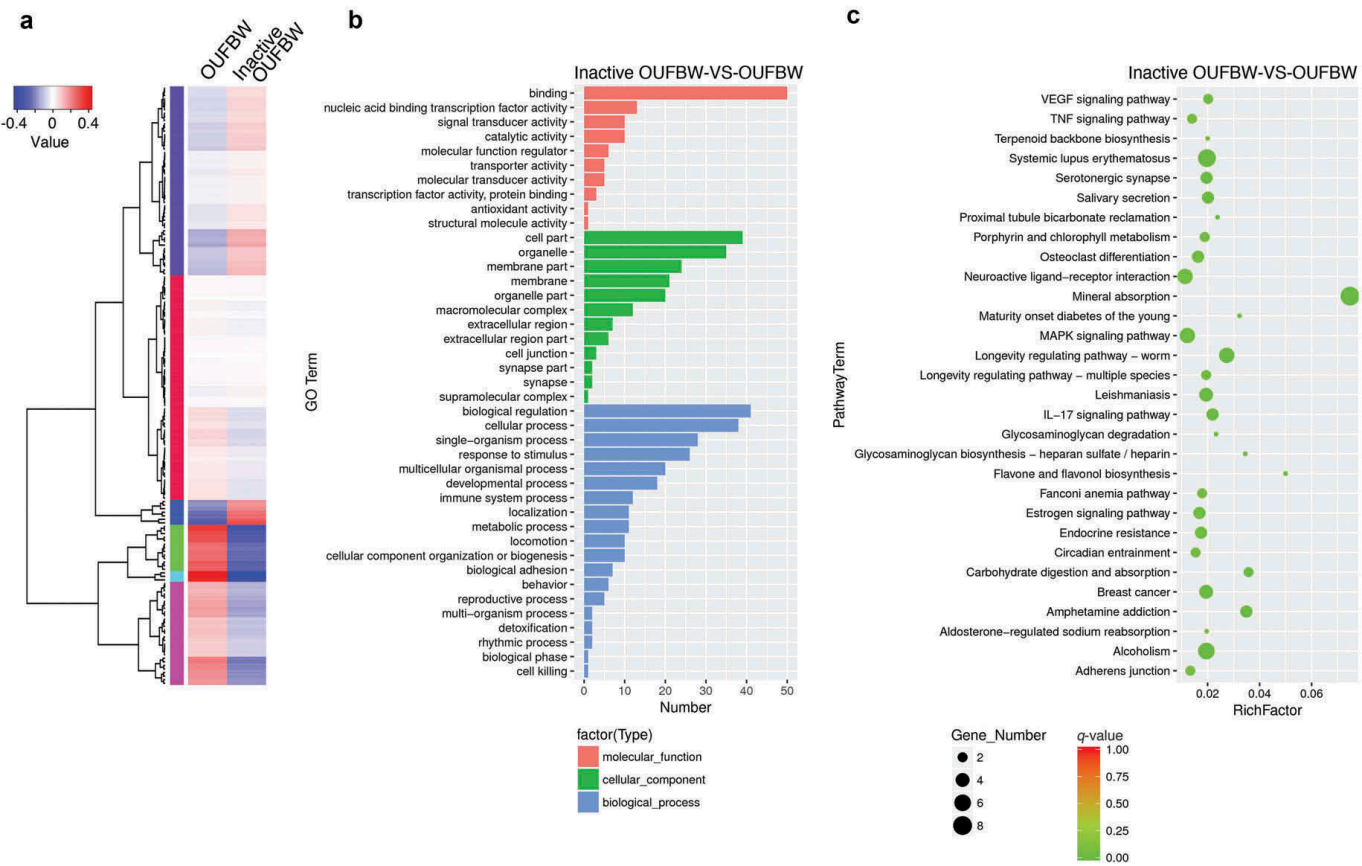
Analysis of the changes of gene expression in hPDLFs by OUFBW treatment using RNA-seq. (a) Cluster analysis of differentially expressed genes (DEGs). The expression levels were visualized and the scale from the abundant to high range is from −0.4 to 0.4. The left trees show the phylogenetic relationships and the regions of different colors represent different clusters. (b) GO enrichment histogram of the DEGs. The distributions are summarized in three main categories: molecular function, cellular component and biological process. The x-axis indicates different GO terms and the y-axis indicates the number of genes in each category. (c) Scatter plots of enriched KEGG functional pathways in response to OUFBW. Top 30 enriched pathways are shown in the figure. RichFactor means the ratio of the number of genes differentially expressed in the pathway to the total number of genes in the pathway. *q-*value is corrected *p-*value ranging between 0 and 1; the closer to 0, the more significant the enrichment.

To comprehensively analyze the transcript profiles of hPDLFs after OUFBW stimulation, we performed RNA sequencing (RNA-seq) using Illumina HiSeq platform. To identify differentially expressed genes (DEGs), we analyzed gene expression levels using edgeR software. The DEGs were identified based on the *p*-value of genes <0.05 and the absolute value of fold change ≥2. To overview this DEGs, hierarchical clustering was constructed with normalized FPKM values to characterize the changes in two samples (Figure 5(a)). In total 88 genes were identified as significantly up-regulated and 80 genes were identified as significantly down-regulated in OUFBW-treated cells compared to inactive OUFBW (Supplementary Table S1). The most up-regulated gene in OUFBW-treated cells was metallothionein-1G (*MT1G*, 59.5-fold), a member of intracellular cysteine-rich, metal-binding proteins. Metallothioneins are involved in the array of protective stress responses against various stimuli including oxidative stress [18]. Also, transcription factor HES1 (*HES1*, 13.9-fold) and c-Fos (*FOS*, 10.2-fold) were up-regulated in OUFBW-treated cells.

GO (http://www.geneontology.org/) is an international standard classification system for gene function and is generally applied to search for enriched GO terms in DEGs. We performed GO enrichment analysis to characterize the up-regulated genes in OUFBW-treated cells. The GO classification of DEGs is available in Supplementary Table S1. The GO terms under the molecular function category were concentrated in ‘binding’. The highest number of GO term under cellular component category was ‘cell part’, while ‘biological regulation’ under biological process category (Figure 5(b)).

KEGG database is a collection of various pathways including the molecular interactions and reaction networks. To specify the signaling pathways involved in OUFBW-treated cells, we performed KEGG enrichment analysis and top 30 pathways that are significantly enriched (*q-*value <0.05) were identified (Figure 5(c) and Supplementary Table S2). DEGs were highly clustered in several signaling pathways such as ‘Mineral absorption’, ‘Neuroactive ligand-receptor interaction’ and ‘MAPK signaling pathway’.

## 4. Discussion

Ozone has been widely used as an antiseptic in the food industry and in the dental fields. The recently developed technology of micro-bubble generation enabled the production of OUFBW, wherein the ozone moiety is extremely stable in solution [10]. A randomized controlled trial demonstrated the clinical efficacy of OUFBW in the treatment of periodontitis [11]. They also showed, using 3D human buccal and gingival tissue models, that OUFBW reduced concentration of periodontitis-related bacteria *Porphyromonas gingivalis* and *Aggregatibacter actinomycetemcomitans* without affecting oral tissues [12]. The low cytotoxicity of OUFBW was confirmed in our study at the cellular level. On the other hand, oxidative stress induced by ozone via the production of ROS is predicted to trigger some cellular signaling that possibly leads to regeneration of periodontal tissues. However, how the ozone moiety in OUFBW triggers cell signaling in the periodontal tissues remains to be elucidated. In this study, we demonstrated that OUFBW induced the production of ROS. The oxidative stress caused by OUFBW induced activation of the MAPK pathway, especially the activation of p38 MAPK. OUFBW triggered the activation of an AP-1 component c-Fos and Nrf2, a transcription factor involved in the induction of oxidative stress. The results of RNA-seq analysis also revealed that the numerous genes involved in oxidative stress responses or MAPK signaling pathway were up-regulated after OUFBW treatment. The investigation of the signaling induced by OUFBW serves to highlight the other biological roles of OUFBW, in addition to its bactericidal activity, in the treatment of periodontitis. In addition to its conventional antiseptic role, our data suggest that the ozone in OUFBW stimulates cellular signaling via eliciting oxidative stress responses.

When hPDLFs or the Ca9-22 human oral epithelial cell line was exposed to OUFBW, production of ROS in the cells was actually induced in an ozone-dependent manner, similar to the observation in the H_2_O_2_-treated cells. In general, the cellular protection against oxidative stress was induced by variety of stimuli such as H_2_O_2_, MAPK pathways, including p38 MAPK, SAPK/JNK and Erk1/2 [14,15]. p38 MAPK is also involved in the induction of hyperresponsiveness and inflammation in the airways after inhalation of gaseous ozone [19]. However, how aqueous ozone affects the MAPK in the cells is still unknown. Therefore, we examined activation of the MAPK pathways after treatment with OUFBW. It was revealed that p38 MAPK was predominantly activated after OUFBW exposure, suggesting that the cellular MAPK signaling is activated through a p38 MAPK-dependent pathway.

Two transcription factors, c-Fos and Nrf2, are known to be involved in the cellular responses to oxidative stress [14,16,17]. Our data showed that both c-Fos and Nrf2 are translocated to the nuclei after treatment with OUFBW, suggesting that these factors trigger the expression of downstream genes in the cells. The transcription factors c-Fos and c-Jun are involved in mechanical stretch-inducing differentiation of osteoblastic cells [20]. The MAPK pathways are also associated with bone morphogenetic protein-9 (BMP-9)–mediated differentiation of hPDLFs to osteoblastic-like cells [21]. Therefore, we speculate that the downstream genes under the control by these transcription factors may play a role in regenerating periodontal tissues.

In RNA-seq analysis, we identified the several differentially expressed genes in OUFBW-treated cells. The most up-regulated metallothionein-1G is a member of intracellular cysteine-rich, metal-binding proteins. Metallothioneins are involved in the array of protective stress responses against various stimuli including oxidative stress [18]. The genes coding metallothioneins have many response elements for up-regulation of transcription in its promoter region that includes ARE. The transcription factor Nrf2 can bind ARE to induce cytoprotective responses to oxidative stress [16]. Therefore, the results suggest the possibility of Nrf2-mediated up-regulation of metallothioneins, which may play a role in protective stress responses against OUFBW stimulation. On the other hand, transcription factor HES1 has been reported to cooperate with retinoblastoma protein to activate transcription factor RUNX2, which is required for osteoblast differentiation and bone formation [22,23]. It may be possible that up-regulated HES1 is involved in osteoblast differentiation in periodontal ligaments. Furthermore, the c-Fos up-regulation in RNA-seq analysis is consistent with the activation MAPK pathway and the nuclear translocation of c-Fos in our study.

Although the precise functions of the genes that are up-regulated must await further study, up-regulated genes by OUFBW treatment may be important in regeneration of periodontal tissues. Our study provides new insight into the molecular mechanisms underlying the cellular responses to ozone in OUFBW.

## 5. Conclusions

Ozone has been widely used as an antiseptic in the food industry and in the dental fields, but it remains unclear how the ozone moiety in OUFBW triggers cell signaling in the periodontal tissues. We provide evidence that cellular signaling including the oxidative stress responses or MAPK pathway is triggered through ROS production stimulated by ozone. Our study hopefully brings a new insight into the molecular mechanisms underlying the cellular responses to ozone in OUFBW.
