# Supplementary material for: Ozone ultrafine bubble water induces the cellular signaling involved in oxidative stress responses in human periodontal ligament fibroblasts
Source: Sci Technol Adv Mater. 2019 Jun 13;20(1):589–98. doi: 10.1080/14686996.2019.1614980 (PMC6586087; doi:10.1080/14686996.2019.1614980)
Supplement: Supplemental Material [file TSTA_A_1614980_SM5109.zip › suppl1.pdf]

Table S1

gene_id	length	OUFBW-inactive.rawreads	OUFBW-inactive.FPKM	OUFBW.rawreads	OUFBW.FPKM	logFC	logCPM	pval	FDR	Regulation	GeneSymbol	KO	GO
ENSG00000125144	750	2	0.06	70	3.57	5.695342194	0.416125623	5.30E-08	0.001525898	Ups	MT1G	K14739	molecular_function:protein binding(GO:0005515);cellular_component:nucleus(GO:0005634);cellular_component:cytoplasm(GO:0005737);molecular_function:zinc ion binding(GO:0008270);biological_process:monocyte differentiation(GO:0030224);biological_process:cellular response to vascular endothelial growth factor stimulus(GO:0035924);biological_process:monocyte activation(GO:0042117);biological_process:negative regulation of growth(GO:0045926);molecular_function:metal ion binding(GO:0046872);cellular_component:perinuclear region of cytoplasm(GO:0048471);biological_process:cellular response to cadmium ion(GO:0071276);biological_process:cellular response to copper ion(GO:0071280);biological_process:cellular response to zinc ion(GO:0071294)
ENSG00000114315	2062	82	0.97	730	13.53	3.822479913	3.905134962	2.84E-07	0.004087312	Ups	HES1	K06054	biological_process:negative regulation of transcription from RNA polymerase II promoter(GO:0000122);molecular_function:RNA polymerase II core promoter proximal region sequence-specific DNA binding transcription factor activity involved in negative regulation of transcription(GO:0001078);biological_process:liver development(GO:0001889);biological_process:embryonic heart tube morphogenesis(GO:0003143);biological_process:outflow tract morphogenesis(GO:0003151);biological_process:regulation of secondary heart field cardioblast proliferation(GO:0003266);biological_process:ventricular septum development(GO:0003281);molecular_function:DNA binding(GO:0003677);molecular_function:sequence-specific DNA binding transcription factor activity(GO:0003700);molecular_function:protein binding(GO:0005515);cellular_component:nucleus(GO:0005634);cellular_component:nucleoplasm(GO:0005654);cellular_component:cytoplasm(GO:0005737);biological_process:protein complex assembly(GO:0006461);biological_process:cell adhesion(GO:0007155);biological_process:Notch signaling pathway(GO:0007219);biological_process:smoothened signaling pathway(GO:0007224);biological_process:STAT protein import into nucleus(GO:0007262);biological_process:nervous system development(GO:0007399);molecular_function:transcription factor binding(GO:0008134);biological_process:positive regulation of cell proliferation(GO:0008284);biological_process:cell migration(GO:0016477);biological_process:telencephalon development(GO:0021537);biological_process:midbrain-hindbrain boundary morphogenesis(GO:0021555);biological_process:oculomotor nerve development(GO:0021557);biological_process:trochlear nerve development(GO:0021558);biological_process:hindbrain morphogenesis(GO:0021575);biological_process:forebrain radial glial cell differentiation(GO:0021861);biological_process:adenohypophysis development(GO:0021984);biological_process:lung development(GO:0030324);biological_process:positive regulation of BMP signaling pathway(GO:0030513);biological_process:midbrain development(GO:0030901);biological_process:endocrine pancreas development(GO:0031018);biological_process:somatic stem cell maintenance(GO:0035019);biological_process:ascending aorta morphogenesis(GO:0035910);biological_process:positive regulation of T cell proliferation(GO:0042102);biological_process:positive regulation of tyrosine phosphorylation of Stat3 protein(GO:0042517);biological_process:auditory receptor cell fate determination(GO:0042668);molecular_function:protein homodimerization activity(GO:0042803);molecular_function:histone deacetylase binding(GO:0042826);biological_process:positive regulation of DNA binding(GO:0043388);molecular_function:sequence-specific DNA binding(GO:0043565);biological_process:regulation of fat cell differentiation(GO:0045598);biological_process:negative regulation of auditory receptor cell differentiation(GO:0045608);biological_process:positive regulation of Notch signaling pathway(GO:0045747);biological_process:negative regulation of transcription, DNA-templated(GO:0045892);biological_process:positive regulation of transcription from RNA polymerase II promoter(GO:0045944);biological_process:positive regulation of mitotic cell cycle, embryonic(GO:0045977);biological_process:lateral inhibition(GO:0046331);biological_process:positive regulation of JAK-STAT cascade(GO:0046427);biological_process:cell maturation(GO:0048469);biological_process:thymus development(GO:0048538);biological_process:cell morphogenesis involved in neuron differentiation(GO:0048667);biological_process:positive regulation of astrocyte differentiation(GO:0048711);biological_process:negative regulation of oligodendrocyte differentiation(GO:0048715);biological_process:artery morphogenesis(GO:0048844);biological_process:pharyngeal system development(GO:0060037);biological_process:regulation of timing of neuron differentiation(GO:0060164);biological_process:negative regulation of glial cell proliferation(GO:0060253);biological_process:ventricular septum morphogenesis(GO:0060412);biological_process:ureteric bud morphogenesis(GO:0060675);biological_process:labyrinthine layer blood vessel development(GO:0060716);biological_process:common bile duct development(GO:0061009);biological_process:negative regulation of stomach neuroendocrine cell differentiation(GO:0061106);biological_process:cardiac neural crest cell development involved in outflow tract morphogenesis(GO:0061309);molecular_function:N-box binding(GO:0071820);biological_process:glomerulus vasculature development(GO:0072012);biological_process:comma-shaped body morphogenesis(GO:0072049);biological_process:S-shaped body morphogenesis(GO:0072050);biological_process:renal interstitial fibroblast development(GO:0072141);biological_process:metanephric nephron tubule morphogenesis(GO:0072282);biological_process:cochlea development(GO:0090102);biological_process:vascular smooth muscle cell development(GO:0097084);biological_process:neuronal stem cell maintenance(GO:0097150);biological_process:negative regulation of pancreatic A cell differentiation(GO:2000227);biological_process:negative regulation of stem cell differentiation(GO:2000737);biological_process:negative regulation of pro-B cell differentiation(GO:2000974);biological_process:negative regulation of forebrain neuron differentiation(GO:2000978)

ENSG00000170345	3238	145	1.09	945	11.15	3.37366936	4.313735946	3.27E-06	0.031357195	Ups	FOS	K04379	biological_process:conditioned taste aversion(GO:0001661);biological_process:toll-like receptor signaling pathway(GO:0002224);biological_process:MyD88-dependent toll-like receptor signaling pathway(GO:0002755);biological_process:MyD88-independent toll-like receptor signaling pathway(GO:0002756);molecular_function:DNA binding(GO:0003677);molecular_function:double-stranded DNA binding(GO:0003690);molecular_function:sequence-specific DNA binding transcription factor activity(GO:0003700);molecular_function:protein binding(GO:0005515);cellular_component:nucleus(GO:0005634);cellular_component:nucleoplasm(GO:0005654);cellular_component:transcription factor complex(GO:0005667);cellular_component:nucleolus(GO:0005730);cellular_component:endoplasmic reticulum(GO:0005783);cellular_component:cytosol(GO:0005829);biological_process:DNA methylation(GO:0006306);biological_process:regulation of transcription from RNA polymerase II promoter(GO:0006357);biological_process:transcription from RNA polymerase II promoter(GO:0006366);biological_process:inflammatory response(GO:0006954);biological_process:transforming growth factor beta receptor signaling pathway(GO:0007179);biological_process:nervous system development(GO:0007399);biological_process:female pregnancy(GO:0007565);biological_process:aging(GO:0007568);molecular_function:transcription factor binding(GO:0008134);biological_process:response to cold(GO:0009409);biological_process:response to light stimulus(GO:0009416);biological_process:response to mechanical stimulus(GO:0009612);biological_process:response to gravity(GO:0009629);biological_process:response to toxic substance(GO:0009636);cellular_component:membrane(GO:0016020);biological_process:sleep(GO:0030431);biological_process:cellular response to extracellular stimulus(GO:0031668);biological_process:response to lipopolysaccharide(GO:0032496);biological_process:response to progesterone(GO:0032570);biological_process:cellular response to hormone stimulus(GO:0032870);biological_process:response to cytokine(GO:0034097);biological_process:toll-like receptor 2 signaling pathway(GO:0034134);biological_process:toll-like receptor 3 signaling pathway(GO:0034138);biological_process:toll-like receptor 4 signaling pathway(GO:0034142);biological_process:toll-like receptor 5 signaling pathway(GO:0034146);biological_process:toll-like receptor 9 signaling pathway(GO:0034162);biological_process:toll-like receptor 10 signaling pathway(GO:0034166);biological_process:cellular response to reactive oxygen species(GO:0034614);biological_process:TRIF-dependent toll-like receptor signaling pathway(GO:0035666);biological_process:skeletal muscle cell differentiation(GO:0035914);biological_process:Fc-epsilon receptor signaling pathway(GO:0038095);biological_process:toll-like receptor TLR1:TLR2 signaling pathway(GO:0038123);biological_process:toll-like receptor TLR6:TLR2 signaling pathway(GO:0038124);biological_process:response to drug(GO:0042493);cellular_component:neuron projection(GO:0043005);molecular_function:sequence-specific DNA binding(GO:0043565);molecular_function:transcription regulatory region DNA binding(GO:0044212);biological_process:innate immune response(GO:0045087);biological_process:positive regulation of transcription, DNA-templated(GO:0045893);biological_process:positive regulation of transcription from RNA polymerase II promoter(GO:0045944);biological_process:regulation of sequence-specific DNA binding transcription factor activity(GO:0051090);biological_process:stress-activated MAPK cascade(GO:0051403);biological_process:response to corticosterone(GO:0051412);biological_process:response to cAMP(GO:0051591);biological_process:SMAD protein signal transduction(GO:0060395);molecular_function:R-SMAD binding(GO:0070412);biological_process:cellular response to calcium ion(GO:0071277)
ENSG00000120738	3138	844	6.55	4069	49.55	2.939918748	6.467158103	2.98E-05	0.214602353	Ups	EGR1	K09203	biological_process:negative regulation of transcription from RNA polymerase II promoter(GO:0000122);molecular_function:transcription regulatory region sequence-specific DNA binding(GO:0000976);molecular_function:RNA polymerase II core promoter sequence-specific DNA binding(GO:0000979);molecular_function:RNA polymerase II core promoter proximal region sequence-specific DNA binding transcription factor activity involved in positive regulation of transcription(GO:0001077);biological_process:response to amphetamine(GO:0001975);molecular_function:DNA binding(GO:0003677);molecular_function:double-stranded DNA binding(GO:0003690);molecular_function:sequence-specific DNA binding transcription factor activity(GO:0003700);molecular_function:protein binding(GO:0005515);cellular_component:nucleus(GO:0005634);cellular_component:nucleoplasm(GO:0005654);cellular_component:cytoplasm(GO:0005737);biological_process:transcription from RNA polymerase II promoter(GO:0006366);biological_process:learning or memory(GO:0007611);biological_process:circadian rhythm(GO:0007623);molecular_function:transcription factor binding(GO:0008134);biological_process:response to glucose(GO:0009749);biological_process:cytokine-mediated signaling pathway(GO:0019221);biological_process:T cell differentiation(GO:0030217);biological_process:BMP signaling pathway(GO:0030509);biological_process:response to nutrient levels(GO:0031667);biological_process:cellular response to insulin stimulus(GO:0032869);biological_process:regulation of protein sumoylation(GO:0033233);biological_process:response to carbon monoxide(GO:0034465);molecular_function:histone acetyltransferase binding(GO:0035035);biological_process:cellular response to drug(GO:0035690);biological_process:skeletal muscle cell differentiation(GO:0035914);biological_process:response to cocaine(GO:0042220);biological_process:positive regulation of neuron apoptotic process(GO:0043525);molecular_function:sequence-specific DNA binding(GO:0043565);biological_process:response to ethanol(GO:0045471);biological_process:positive regulation of transcription, DNA-templated(GO:0045893);biological_process:positive regulation of transcription from RNA polymerase II promoter(GO:0045944);molecular_function:metal ion binding(GO:0046872);biological_process:regulation of long-term neuronal synaptic plasticity(GO:0048169);biological_process:oligodendrocyte differentiation(GO:0048709);biological_process:response to electrical stimulus(GO:0051602);biological_process:long-term synaptic potentiation(GO:0060291);biological_process:type I interferon signaling pathway(GO:0060337);biological_process:interleukin-1-mediated signaling pathway(GO:0070498);biological_process:cellular response to antibiotic(GO:0071236);biological_process:cellular response to mechanical stimulus(GO:0071260);biological_process:cellular response to isoquinoline alkaloid(GO:0071317);biological_process:cellular response to cAMP(GO:0071320);biological_process:cellular response to growth factor stimulus(GO:0071363);biological_process:cellular response to follicle-stimulating hormone stimulus(GO:0071372);biological_process:cellular response to steroid hormone stimulus(GO:0071383);biological_process:cellular response to hyperoxia(GO:0071455);biological_process:cellular response to hypoxia(GO:0071456);biological_process:cellular response to gamma radiation(GO:0071480);biological_process:cellular response to heparin(GO:0071504);biological_process:cellular response to mycophenolic acid(GO:0071506);biological_process:response to norepinephrine(GO:0071873);biological_process:glomerular mesangial cell proliferation(GO:0072110);biological_process:positive regulation of glomerular metanephric mesangial cell proliferation(GO:0072303);biological_process:negative regulation of canonical Wnt signaling pathway(GO:0090090)
ENSG00000260075	1471	118	1.95	7	0.18	-3.386633454	0.765787469	6.12E-05	0.35266858	Down	NSFP1	K06027	–
ENSG00000173110	2646	74	0.68	291	4.2	2.643697263	2.691551767	0.000229544	1	Ups	HSPA6	K03283	molecular_function:ATP binding(GO:0005524);biological_process:response to unfolded protein(GO:0006986);cellular_component:COP9 signalosome(GO:0008180)
ENSG00000174384	613	23	0.91	0	0	-7.236407054	-1.406826257	0.000390825	1	Down	RP11-313P13.4	K10858	–
ENSG00000197846	1195	1	0.02	15	0.48	4.380848251	-1.651477067	0.000530405	1	Ups	HIST1H2BF	K11252	–

ENSG00000163909	3750	6	0.04	34	0.35	3.140919075	-0.464465284	0.000533686	1	Ups	HEYL	K09091	molecular_function:RNA polymerase II core promoter sequence-specific DNA binding(GO:0000979);molecular_function:RNA polymerase II core promoter sequence-specific DNA binding transcription factor activity(GO:0000983);molecular_function:protein binding transcription factor activity(GO:0000988);molecular_function:RNA polymerase II core promoter proximal region sequence-specific DNA binding transcription factor activity involved in positive regulation of transcription(GO:0001077);molecular_function:RNA polymerase II transcription corepressor activity(GO:0001106);biological_process:outflow tract morphogenesis(GO:0003151);biological_process:atrioventricular valve morphogenesis(GO:0003181);biological_process:pulmonary valve morphogenesis(GO:0003184);biological_process:epithelial to mesenchymal transition involved in endocardial cushion formation(GO:0003198);biological_process:endocardial cushion morphogenesis(GO:0003203);biological_process:cardiac ventricle morphogenesis(GO:0003208);molecular_function:sequence-specific DNA binding transcription factor activity(GO:0003700);molecular_function:transcription corepressor activity(GO:0003714);molecular_function:protein binding(GO:0005515);cellular_component:nucleus(GO:0005634);cellular_component:nucleoplasm(GO:0005654);cellular_component:cytoplasm(GO:0005737);biological_process:Notch signaling pathway(GO:0007219);molecular_function:transcription factor binding(GO:0008134);biological_process:mesenchymal cell development(GO:0014031);biological_process:glomerulus development(GO:0032835);biological_process:skeletal muscle cell differentiation(GO:0035914);molecular_function:microsatellite binding(GO:0035939);molecular_function:protein homodimerization activity(GO:0042803);biological_process:positive regulation of neuron differentiation(GO:0045666);biological_process:negative regulation of transcription, DNA-templated(GO:0045892);biological_process:positive regulation of transcription from RNA polymerase II promoter(GO:0045944);molecular_function:protein heterodimerization activity(GO:0046982);molecular_function:AF-1 domain binding(GO:0050683);biological_process:cardiac epithelial to mesenchymal transition(GO:0060317);biological_process:ventricular septum morphogenesis(GO:0060412);biological_process:negative regulation of androgen receptor signaling pathway(GO:0060766);biological_process:cellular response to BMP stimulus(GO:0071773);biological_process:proximal tubule development(GO:0072014);biological_process:negative regulation of androgen receptor activity(GO:2000824)
ENSG00000187764	14160	51	0.09	183	0.49	2.510485266	2.039322926	0.000558575	1	Ups	SEMA4D	K06521	biological_process:negative regulation of transcription from RNA polymerase II promoter(GO:0000122);biological_process:positive regulation of protein phosphorylation(GO:0001934);molecular_function:receptor activity(GO:0004872);molecular_function:transmembrane signaling receptor activity(GO:0004888);molecular_function:receptor binding(GO:0005102);molecular_function:protein binding(GO:0005515);cellular_component:extracellular space(GO:0005615);cellular_component:plasma membrane(GO:0005886);cellular_component:integral component of plasma membrane(GO:0005887);biological_process:immune response(GO:0006955);biological_process:cell adhesion(GO:0007155);biological_process:axon guidance(GO:0007411);biological_process:regulation of cell shape(GO:0008360);biological_process:negative regulation of alkaline phosphatase activity(GO:0010693);biological_process:positive regulation of phosphatidylinositol 3-kinase signaling(GO:0014068);molecular_function:semaphorin receptor binding(GO:0030215);biological_process:positive regulation of cell migration(GO:0030335);biological_process:regulation of cell projection organization(GO:0031344);;biological_process:negative regulation of apoptotic process(GO:0043066);biological_process:ossification involved in bone maturation(GO:0043931);biological_process:negative regulation of osteoblast differentiation(GO:0045668);biological_process:positive regulation of collateral sprouting(GO:0048672);biological_process:regulation of dendrite morphogenesis(GO:0048814);biological_process:positive regulation of peptidyl-tyrosine phosphorylation(GO:0050731);biological_process:negative regulation of peptidyl-tyrosine phosphorylation(GO:0050732);biological_process:positive regulation of axonogenesis(GO:0050772);biological_process:leukocyte aggregation(GO:0070486);biological_process:semaphorin-plexin signaling pathway(GO:0071526);biological_process:semaphorin-plexin signaling pathway involved in bone trabecula morphogenesis(GO:1900220)
ENSG00000162772	4003	215	1.31	700	6.68	2.372974687	4.003489495	0.000654102	1	Ups	ATF3	K09032	molecular_function:sequence-specific DNA binding transcription factor activity(GO:0003700);molecular_function:transcription corepressor activity(GO:0003714);molecular_function:protein binding(GO:0005515);cellular_component:nucleus(GO:0005634);cellular_component:nucleoplasm(GO:0005654);cellular_component:nucleolus(GO:0005730);biological_process:gluconeogenesis(GO:0006094);biological_process:transcription, DNA-templated(GO:0006351);biological_process:response to stress(GO:0006950);biological_process:activation of signaling protein activity involved in unfolded protein response(GO:0006987);biological_process:positive regulation of cell proliferation(GO:0008284);biological_process:endoplasmic reticulum unfolded protein response(GO:0030968);biological_process:skeletal muscle cell differentiation(GO:0035914);molecular_function:identical protein binding(GO:0042802);molecular_function:sequence-specific DNA binding(GO:0043565);biological_process:cellular protein metabolic process(GO:0044267);biological_process:negative regulation of transcription, DNA-templated(GO:0045892)
ENSG00000253295	472	1	0.05	14	1.13	4.281970822	-1.730891932	0.000726851	1	Ups	RP11-779O18.1	_	_
ENSG00000225217	1927	14	0.18	60	1.19	2.756903782	0.390351053	0.000799282	1	Ups	HSPA7	K03283	molecular_function:ATP binding(GO:0005524);biological_process:response to unfolded protein(GO:0006986);cellular_component:COP9 signalosome(GO:0008180)
ENSG00000125740	5553	240	1.05	758	5.22	2.329211913	4.125837382	0.000800425	1	Ups	FOSB	K09029	biological_process:negative regulation of transcription from RNA polymerase II promoter(GO:0000122);molecular_function:DNA binding(GO:0003677);molecular_function:sequence-specific DNA binding transcription factor activity(GO:0003700);cellular_component:nucleus(GO:0005634);biological_process:multicellular organismal development(GO:0007275);biological_process:behavior(GO:0007610);molecular_function:transcription factor binding(GO:0008134);molecular_function:sequence-specific DNA binding(GO:0043565);biological_process:cellular response to calcium ion(GO:0071277)
ENSG00000187193	1773	230	3.16	711	15.32	2.298236198	4.038588548	0.000930366	1	Ups	MT1X	K14739	cellular_component:nucleus(GO:0005634);cellular_component:cytoplasm(GO:0005737);biological_process:cellular zinc ion homeostasis(GO:0006882);biological_process:nitric oxide mediated signal transduction(GO:0007263);molecular_function:zinc ion binding(GO:0008270);biological_process:response to metal ion(GO:0010038);biological_process:detoxification of copper ion(GO:0010273);biological_process:cellular response to erythropoietin(GO:0036018);biological_process:negative regulation of growth(GO:0045926);molecular_function:metal ion binding(GO:0046872);cellular_component:perinuclear region of cytoplasm(GO:0048471);biological_process:cellular response to cadmium ion(GO:0071276);biological_process:cellular response to zinc ion(GO:0071294)
ENSG00000137270	2765	0	0	13	0.18	7.085137539	-1.890726543	0.001006409	1	Ups	GCM1	K12581	molecular_function:DNA binding(GO:0003677);molecular_function:sequence-specific DNA binding transcription factor activity(GO:0003700);cellular_component:nucleus(GO:0005634);cellular_component:transcription factor complex(GO:0005667);biological_process:transcription, DNA-templated(GO:0006351);biological_process:regulation of transcription, DNA-templated(GO:0006355);molecular_function:transcription factor binding(GO:0008134);molecular_function:zinc ion binding(GO:0008270);biological_process:anatomical structure morphogenesis(GO:0009653);biological_process:astrocyte fate commitment(GO:0060018);biological_process:branching involved in labyrinthine layer morphogenesis(GO:0060670);biological_process:cell differentiation involved in embryonic placenta development(GO:0060706)
ENSG00000267793	570	33	1.41	1	0.07	-4.247460031	-0.930039484	0.001141053	1	Down	RP11-576C2.1	_	_
ENSG00000240687	832	17	0.5	0	0	-6.803680762	-1.752423558	0.001996045	1	Down	RP11-521D12.1	_	_
ENSG00000188693	2957	16	0.13	0	0	-6.717024823	-1.819680444	0.002863891	1	Down	CTB-161K23.1	_	_
ENSG00000254101	2706	16	0.14	0	0	-6.717024823	-1.819680444	0.002863891	1	Down	RP11-30J20.1	_	_

ENSG00000073756	5269	1446	6.69	3502	25.4	1.946785694	6.407073818	0.004068568	1 Ups	PTGS2	K11987	biological_process:prostaglandin biosynthetic process(GO:0001516);biological_process:angiogenesis(GO:0001525);molecular_function:peroxidase activity(GO:0004601);molecular_function:prostaglandin-endoperoxide synthase activity(GO:0004666);cellular_component:nucleus(GO:0005634);cellular_component:cytoplasm(GO:0005737);cellular_component:endoplasmic reticulum membrane(GO:0005789);biological_process:prostaglandin metabolic process(GO:0006693);biological_process:movement of cell or subcellular component(GO:0006928);biological_process:inflammatory response(GO:0006954);biological_process:response to oxidative stress(GO:0006979);biological_process:embryo implantation(GO:0007566);biological_process:learning(GO:0007612);biological_process:memory(GO:0007613);biological_process:regulation of blood pressure(GO:0008217);biological_process:negative regulation of cell proliferation(GO:0008285);molecular_function:lipid binding(GO:0008289);biological_process:response to fructose(GO:0009750);biological_process:response to manganese ion(GO:0010042);biological_process:response to lithium ion(GO:0010226);biological_process:positive regulation of vascular endothelial growth factor production(GO:0010575);biological_process:sensory perception of pain(GO:0019233);biological_process:arachidonic acid metabolic process(GO:0019369);biological_process:cyclooxygenase pathway(GO:0019371);biological_process:lipoxygenase pathway(GO:0019372);molecular_function:enzyme binding(GO:0019899);molecular_function:heme binding(GO:0020037);biological_process:bone mineralization(GO:0030282);biological_process:ovulation(GO:0030728);biological_process:positive regulation of prostaglandin biosynthetic process(GO:0031394);biological_process:positive regulation of fever generation(GO:0031622);biological_process:positive regulation of synaptic plasticity(GO:0031915);biological_process:negative regulation of synaptic transmission, dopaminergic(GO:0032227);biological_process:response to estradiol(GO:0032355);biological_process:response to lipopolysaccharide(GO:0032496);biological_process:response to vitamin D(GO:0033280);biological_process:response to tumor necrosis factor(GO:0034612);biological_process:cellular response to UV(GO:0034644);biological_process:maintenance of blood-brain barrier(GO:0035633);biological_process:positive regulation of NF-kappaB import into nucleus(GO:0042346);biological_process:response to drug(GO:0042493);biological_process:anagen(GO:0042640);molecular_function:protein homodimerization activity(GO:0042803);cellular_component:neuron projection(GO:0043005);biological_process:positive regulation of apoptotic process(GO:0043065);cellular_component:protein complex(GO:0043234);biological_process:small molecule metabolic process(GO:0044281);biological_process:positive regulation of nitric oxide biosynthetic process(GO:0045429);biological_process:negative regulation of cell cycle(GO:0045786);biological_process:positive regulation of vasoconstriction(GO:0045907);biological_process:negative regulation of smooth muscle contraction(GO:0045986);biological_process:positive regulation of smooth muscle contraction(GO:0045987);biological_process:decidualization(GO:0046697);molecular_function:metal ion binding(GO:0046872);biological_process:positive regulation of smooth muscle cell proliferation(GO:0048661);molecular_function:arachidonate 15-lipoxygenase activity(GO:0050473);biological_process:regulation of inflammatory response(GO:0050727);biological_process:brown fat cell differentiation(GO:0050873);biological_process:response to glucocorticoid(GO:0051384);biological_process:negative regulation of calcium ion transport(GO:0051926);biological_process:positive regulation of synaptic transmission, glutamatergic(GO:0051968);biological_process:response to fatty acid(GO:0070542);biological_process:cellular response to mechanical stimulus(GO:0071260);biological_process:cellular response to ATP(GO:0071318);biological_process:cellular response to hypoxia(GO:0071456);biological_process:positive regulation of transforming growth factor beta production(GO:0071636);biological_process:positive regulation of cell migration involved in sprouting angiogenesis(GO:0090050);biological_process:positive regulation of fibroblast growth factor production(GO:0090271);biological_process:positive regulation of brown fat cell differentiation(GO:0090336);biological_process:positive regulation of platelet-derived growth factor production(GO:0090362)
ENSG00000228327	1317	26	0.48	1	0.03	-3.905303427	-1.207981158	0.004550577	1 Down	RP11-206L10.2	_	_
ENSG00000237927	2002	3	0.04	17	0.32	3.109437198	-1.389914718	0.004550577	1 Ups	RP3-393E18.2	_	_
ENSG00000176399	5569	13	0.06	0	0	-6.420625731	-2.04413534	0.006149306	1 Down	DMRTA1	K19491	molecular_function:sequence-specific DNA binding transcription factor activity(GO:0003700);cellular_component:nucleus(GO:0005634);biological_process:transcription, DNA-templated(GO:0006351);molecular_function:sequence-specific DNA binding(GO:0043565);molecular_function:metal ion binding(GO:0046872)
ENSG00000197748	7184	13	0.04	0	0	-6.420625731	-2.04413534	0.006149306	1 Down	WDR96	_	_
ENSG00000227200	373	13	0.85	0	0	-6.420625731	-2.04413534	0.006149306	1 Down	RP11-121A14.3	_	_
ENSG00000237533	399	13	0.79	0	0	-6.420625731	-2.04413534	0.006149306	1 Down	PFN1P6	K05759	_
ENSG00000198576	3991	174	1.06	402	3.85	1.877969367	3.296237488	0.006299067	1 Ups	ARC	K15867	cellular_component:acrosomal vesicle(GO:0001669);cellular_component:cytoplasm(GO:0005737);cellular_component:endosome(GO:0005768);cellular_component:plasma membrane(GO:0005886);biological_process:endocytosis(GO:0006897);biological_process:cytoskeleton organization(GO:0007010);biological_process:endoderm development(GO:0007492);biological_process:learning(GO:0007612);biological_process:anterior/posterior pattern specification(GO:0009952);cellular_component:postsynaptic density(GO:0014069);cellular_component:actin cytoskeleton(GO:0015629);biological_process:cell migration(GO:0016477);biological_process:regulation of cell morphogenesis(GO:0022604);cellular_component:cell junction(GO:0030054);cellular_component:dendritic spine(GO:0043197);cellular_component:postsynaptic membrane(GO:0045211);biological_process:regulation of neuronal synaptic plasticity(GO:0048168)
ENSG00000235636	882	36	0.99	92	3.99	2.019796002	1.136033556	0.006301016	1 Ups	NUS1P1	K19177	_
ENSG00000230567	2695	1	0.01	12	0.17	4.0612227	-1.902763275	0.007447428	1 Ups	FAM203B	_	_
ENSG00000042781	20584	2	0	12	0.02	3.160584803	-1.826300125	0.007447428	1 Ups	USH2A	K19636	cellular_component:stereocilia ankle link complex(GO:0002142);molecular_function:protein binding(GO:0005515);molecular_function:collagen binding(GO:0005518);cellular_component:basement membrane(GO:0005604);cellular_component:cytoplasm(GO:0005737);biological_process:visual perception(GO:0007601);biological_process:sensory perception of sound(GO:0007605);cellular_component:integral component of membrane(GO:0016021);cellular_component:apical plasma membrane(GO:0016324);molecular_function:myosin binding(GO:0017022);cellular_component:stereocilium bundle(GO:0032421);biological_process:hair cell differentiation(GO:0035315);biological_process:photoreceptor cell maintenance(GO:0045494);biological_process:maintenance of organ identity(GO:0048496);biological_process:response to stimulus(GO:0050896);biological_process:sensory perception of light stimulus(GO:0050953);biological_process:inner ear receptor cell differentiation(GO:0060113);cellular_component:stereocilium membrane(GO:0060171)
ENSG00000124107	596	2	0.08	12	0.77	3.160584803	-1.826300125	0.007447428	1 Ups	SLPI	_	molecular_function:endopeptidase inhibitor activity(GO:0004866);molecular_function:serine-type endopeptidase inhibitor activity(GO:0004867);molecular_function:protein binding(GO:0005515);cellular_component:extracellular region(GO:0005576);biological_process:negative regulation of endopeptidase activity(GO:0010951);molecular_function:enzyme binding(GO:0019899);cellular_component:extracellular matrix(GO:0031012);biological_process:negative regulation of protein binding(GO:0032091);biological_process:negative regulation of viral genome replication(GO:0045071)

ENSG00000089692	3056	11	0.09	33	0.41	2.239961606	-0.365235358	0.007680727	1	Ups	LAG3	K06565	molecular_function:antigen binding(GO:0003823);molecular_function:transmembrane signaling receptor activity(GO:0004888);biological_process:cell surface receptor signaling pathway(GO:0007166);cellular_component:external side of plasma membrane(GO:0009897);cellular_component:integral component of membrane(GO:0016021);molecular_function:MHC class II protein binding(GO:0042289);biological_process:negative regulation of interleukin-2 biosynthetic process(GO:0045085);biological_process:positive regulation of natural killer cell mediated cytotoxicity(GO:0045954);biological_process:negative regulation of T cell activation(GO:0050868)
ENSG00000130518	9153	81	0.22	184	0.77	1.852523384	2.172767139	0.008772716	1	Ups	KIAA1683	K09488	cellular_component:mitochondrion(GO:0005739)
ENSG00000170385	5474	1215	5.41	2583	18.03	1.758750506	6.008735046	0.009077493	1	Ups	SLC30A1	K14688	biological_process:in utero embryonic development(GO:0001701);molecular_function:zinc ion transmembrane transporter activity(GO:0005385);cellular_component:plasma membrane(GO:0005886);biological_process:zinc II ion transport(GO:0006829);biological_process:cellular calcium ion homeostasis(GO:0006874);biological_process:cellular zinc ion homeostasis(GO:0006882);cellular_component:integral component of membrane(GO:0016021);molecular_function:calcium channel inhibitor activity(GO:0019855);cellular_component:T-tubule(GO:0030315);biological_process:negative regulation of neurotransmitter secretion(GO:0046929);biological_process:transmembrane transport(GO:0055085);biological_process:calcium ion import(GO:0070509);biological_process:cadmium ion transmembrane transport(GO:0070574);biological_process:negative regulation of zinc ion transmembrane import(GO:0071584);biological_process:detoxification of cadmium ion(GO:0071585);biological_process:negative regulation of calcium ion import(GO:0090281)
ENSG00000243389	425	0	0	8	0.72	6.391323311	-2.424466088	0.009223959	1	Ups	AC012442.5	_	–
ENSG00000262147	318	0	0	8	0.96	6.391323311	-2.424466088	0.009223959	1	Ups	RP13-638C3.3	_	–
ENSG00000230002	2107	12	0.14	0	0	-6.306551267	-2.128038307	0.009223959	1	Down	ALMS1-IT1	_	–
ENSG00000231563	283	12	1.03	0	0	-6.306551267	-2.128038307	0.009223959	1	Down	RP11-245P10.4	_	–
ENSG00000236107	3839	12	0.08	0	0	-6.306551267	-2.128038307	0.009223959	1	Down	AC010127.3	_	–
ENSG00000205364	942	89	2.3	198	8.03	1.822626558	2.285498699	0.009271549	1	Ups	MT1M	K14739	cellular_component:nucleus(GO:0005634);cellular_component:cytoplasm(GO:0005737);molecular_function:zinc ion binding(GO:0008270);biological_process:negative regulation of growth(GO:0045926);molecular_function:metal ion binding(GO:0046872);cellular_component:perinuclear region of cytoplasm(GO:0048471);biological_process:cellular response to zinc ion(GO:0071294)
ENSG00000260475	864	22	0.62	1	0.04	-3.665834515	-1.396922572	0.009774911	1	Down	RP11-85A1.3	_	–
ENSG00000232060	602	70	2.83	10	0.63	-2.125879922	0.202729716	0.009974628	1	Down	RP11-255N24.4	_	–
ENSG00000174912	1045	12	0.28	34	1.24	2.159031823	-0.307393873	0.010570233	1	Ups	METTL15P1	_	molecular_function:methyltransferase activity(GO:0008168)
ENSG00000169715	1052	1197	27.72	2451	89.03	1.704606668	5.945614712	0.011322029	1	Ups	MT1E	K14739	molecular_function:copper ion binding(GO:0005507);cellular_component:nucleus(GO:0005634);cellular_component:cytoplasm(GO:0005737);molecular_function:zinc ion binding(GO:0008270);biological_process:negative regulation of growth(GO:0045926);molecular_function:cadmium ion binding(GO:0046870);molecular_function:metal ion binding(GO:0046872);cellular_component:perinuclear region of cytoplasm(GO:0048471);biological_process:cellular response to cadmium ion(GO:0071276);biological_process:cellular response to zinc ion(GO:0071294)
ENSG00000156427	1986	8	0.1	25	0.48	2.292771331	-0.755910622	0.012065199	1	Ups	FGF18	K04358	biological_process:angiogenesis(GO:0001525);biological_process:intramembranous ossification(GO:0001957);biological_process:endochondral ossification(GO:0001958);biological_process:chondrocyte development(GO:0002063);molecular_function:type 1 fibroblast growth factor receptor binding(GO:0005105);molecular_function:type 2 fibroblast growth factor receptor binding(GO:0005111);cellular_component:extracellular region(GO:0005576);cellular_component:extracellular space(GO:0005615);cellular_component:nucleolus(GO:0005730);biological_process:signal transduction(GO:0007165);biological_process:epidermal growth factor receptor signaling pathway(GO:0007173);biological_process:cell-cell signaling(GO:0007267);biological_process:nervous system development(GO:0007399);molecular_function:growth factor activity(GO:0008083);biological_process:positive regulation of cell proliferation(GO:0008284);biological_process:insulin receptor signaling pathway(GO:0008286);biological_process:fibroblast growth factor receptor signaling pathway(GO:0008543);biological_process:anatomical structure morphogenesis(GO:0009653);biological_process:lung development(GO:0030324);biological_process:positive regulation of vascular endothelial growth factor receptor signaling pathway(GO:0030949);biological_process:positive regulation of chondrocyte differentiation(GO:0032332);biological_process:Fc-epsilon receptor signaling pathway(GO:0038095);biological_process:innate immune response(GO:0045087);biological_process:neurotrophin TRK receptor signaling pathway(GO:0048011);biological_process:phosphatidylinositol-mediated signaling(GO:0048015);biological_process:positive regulation of ERK1 and ERK2 cascade(GO:0070374)
ENSG00000197847	3984	11	0.07	0	0	-6.182676427	-2.217465781	0.014078675	1	Down	SLC22A20	K08216	biological_process:ion transport(GO:0006811);cellular_component:integral component of membrane(GO:0016021);molecular_function:transmembrane transporter activity(GO:0022857)
ENSG00000216109	89	11	3.01	0	0	-6.182676427	-2.217465781	0.014078675	1	Down	AL713999.1	_	–
ENSG00000230583	2706	11	0.1	0	0	-6.182676427	-2.217465781	0.014078675	1	Down	GTF2IRD1P1	K03121	–
ENSG00000231768	2870	11	0.09	0	0	-6.182676427	-2.217465781	0.014078675	1	Down	RP5-855F14.1	_	–
ENSG00000259116	558	11	0.48	0	0	-6.182676427	-2.217465781	0.014078675	1	Down	RP11-973N13.4	_	–
ENSG00000259344	797	11	0.34	0	0	-6.182676427	-2.217465781	0.014078675	1	Down	RP11-566K19.6	K10595	–
ENSG00000234175	338	4	0.29	16	1.81	2.625114549	-1.400583372	0.014413706	1	Ups	RP11-730A19.9	_	–
ENSG00000268947	1016	29	0.7	3	0.11	-2.564195464	-0.986145374	0.015514096	1	Down	AD000684.2	_	–
ENSG00000139193	1410	28	0.48	3	0.08	-2.513840739	-1.024372606	0.015514096	1	Down	CD27	K05144	molecular_function:transmembrane signaling receptor activity(GO:0004888);molecular_function:protein binding(GO:0005515);cellular_component:extracellular region(GO:0005576);cellular_component:integral component of plasma membrane(GO:0005887);;biological_process:cell surface receptor signaling pathway(GO:0007166);biological_process:release of cytoplasmic sequestered NF-kappaB(GO:0008588);cellular_component:external side of plasma membrane(GO:0009897);biological_process:immunoglobulin mediated immune response(GO:0016064);molecular_function:cysteine-type endopeptidase inhibitor activity involved in apoptotic process(GO:0043027);biological_process:negative regulation of apoptotic process(GO:0043066);biological_process:negative regulation of cysteine-type endopeptidase activity involved in apoptotic process(GO:0043154);biological_process:response to ethanol(GO:0045471);biological_process:positive regulation of B cell differentiation(GO:0045579);biological_process:positive regulation of JNK cascade(GO:0046330);biological_process:extrinsic apoptotic signaling pathway(GO:0097191)
ENSG00000198208	7617	28	0.09	3	0.02	-2.513840739	-1.024372606	0.015514096	1	Down	RPS6KL1	K17985	molecular_function:protein kinase activity(GO:0004672);molecular_function:protein serine/threonine kinase activity(GO:0004674);molecular_function:ATP binding(GO:0005524);cellular_component:ribosome(GO:0005840)

ENSG00000183114	2581	3	0.03	13	0.19	2.724916282	-1.674287139	0.016782554	1 Ups	FAM43B	K15688	–
ENSG00000077522	5419	6	0.03	18	0.13	2.22700435	-1.178824567	0.019112419	1 Ups	ACTN2	K05699	biological_process:platelet degranulation(GO:0002576);molecular_function:actin binding(GO:0003779);molecular_function:integrin binding(GO:0005178);molecular_function:calcium ion binding(GO:0005509);molecular_function:protein binding(GO:0005515);cellular_component:extracellular region(GO:0005576);cellular_component:cytosol(GO:0005829);cellular_component:cytoskeleton(GO:0005856);cellular_component:actin filament(GO:0005884);cellular_component:focal adhesion(GO:0005925);biological_process:muscle contraction(GO:0006936);biological_process:cell adhesion(GO:0007155);biological_process:synaptic transmission(GO:0007268);biological_process:blood coagulation(GO:0007596);molecular_function:cytoskeletal protein binding(GO:0008092);molecular_function:structural constituent of muscle(GO:0008307);cellular_component:Z disc(GO:0030018);biological_process:microspike assembly(GO:0030035);biological_process:muscle filament sliding(GO:0030049);biological_process:platelet activation(GO:0030168);cellular_component:filopodium(GO:0030175);molecular_function:LIM domain binding(GO:0030274);molecular_function:thyroid hormone receptor coactivator activity(GO:0030375);cellular_component:platelet alpha granule lumen(GO:0031093);cellular_component:pseudopodium(GO:0031143);molecular_function:titin binding(GO:0031432);biological_process:regulation of membrane potential(GO:0042391);molecular_function:identical protein binding(GO:0042802);biological_process:regulation of apoptotic process(GO:0042981);cellular_component:dendritic spine(GO:0043197);biological_process:negative regulation of potassium ion transport(GO:0043267);biological_process:positive regulation of potassium ion transport(GO:0043268);molecular_function:ion channel binding(GO:0044325);molecular_function:protein dimerization activity(GO:0046983);biological_process:focal adhesion assembly(GO:0048041);molecular_function:actin filament binding(GO:0051015);biological_process:actin filament bundle assembly(GO:0051017);biological_process:protein homotetramerization(GO:0051289);molecular_function:FATZ binding(GO:0051373);biological_process:actin crosslink formation(GO:0051764);molecular_function:titin Z domain binding(GO:0070080);biological_process:negative regulation of potassium ion transmembrane transporter activity(GO:1901017);biological_process:positive regulation of potassium ion transmembrane transporter activity(GO:1901018);biological_process:negative regulation of protein localization to cell surface(GO:2000009)
ENSG00000111679	5009	6	0.03	18	0.14	2.22700435	-1.178824567	0.019112419	1 Ups	PTPN6	K05697	molecular_function:phosphotyrosine binding(GO:0001784);biological_process:negative regulation of humoral immune response mediated by circulating immunoglobulin(GO:0002924);molecular_function:protein tyrosine phosphatase activity(GO:0004725);molecular_function:transmembrane receptor protein tyrosine phosphatase activity(GO:0005001);molecular_function:protein binding(GO:0005515);cellular_component:nucleus(GO:0005634);cellular_component:cytoplasm(GO:0005737);cellular_component:cytosol(GO:0005829);biological_process:protein dephosphorylation(GO:0006470);biological_process:apoptotic process(GO:0006915);biological_process:G-protein coupled receptor signaling pathway(GO:0007186);biological_process:blood coagulation(GO:0007596);biological_process:cell proliferation(GO:0008283);biological_process:positive regulation of cell proliferation(GO:0008284);biological_process:negative regulation of cell proliferation(GO:0008285);biological_process:positive regulation of phosphatidylinositol 3-kinase signaling(GO:0014068);cellular_component:membrane(GO:0016020);molecular_function:SH3 domain binding(GO:0017124);biological_process:peptidyl-tyrosine phosphorylation(GO:0018108);biological_process:cytokine-mediated signaling pathway(GO:0019221);molecular_function:protein kinase binding(GO:0019901);biological_process:cell differentiation(GO:0030154);biological_process:T cell costimulation(GO:0031295);biological_process:intracellular signal transduction(GO:0035556);cellular_component:alpha-beta T cell receptor complex(GO:0042105);biological_process:negative regulation of T cell proliferation(GO:0042130);molecular_function:SH2 domain binding(GO:0042169);biological_process:natural killer cell mediated cytotoxicity(GO:0042267);biological_process:negative regulation of MAP kinase activity(GO:0043407);biological_process:regulation of B cell differentiation(GO:0045577);biological_process:negative regulation of peptidyl-tyrosine phosphorylation(GO:0050732);biological_process:B cell receptor signaling pathway(GO:0050853);biological_process:negative regulation of T cell receptor signaling pathway(GO:0050860);biological_process:leukocyte migration(GO:0050900);biological_process:interferon-gamma-mediated signaling pathway(GO:0060333);biological_process:regulation of interferon-gamma-mediated signaling pathway(GO:0060334);biological_process:type I interferon signaling pathway(GO:0060337);biological_process:regulation of type I interferon-mediated signaling pathway(GO:0060338);biological_process:JAK-STAT cascade involved in growth hormone signaling pathway(GO:0060397);biological_process:regulation of ERK1 and ERK2 cascade(GO:0070372);biological_process:regulation of G1/S transition of mitotic cell cycle(GO:2000045)
ENSG00000175591	2861	1	0.01	10	0.13	3.800487168	-2.095571103	0.019786881	1 Ups	P2RY2	K04269	molecular_function:receptor activity(GO:0004872);cellular_component:plasma membrane(GO:0005886);cellular_component:integral component of plasma membrane(GO:0005887);biological_process:cellular ion homeostasis(GO:0006873);biological_process:phospholipase C-activating G-protein coupled receptor signaling pathway(GO:0007200);biological_process:regulation of vasodilation(GO:0042312);molecular_function:G-protein coupled purinergic nucleotide receptor activity(GO:0045028);biological_process:positive regulation of mucus secretion(GO:0070257)
ENSG00000235214	352	1	0.07	10	1.09	3.800487168	-2.095571103	0.019786881	1 Ups	FAM83C-AS1	_	–
ENSG00000142583	6854	2	0.01	10	0.06	2.899849271	-2.008436072	0.019786881	1 Ups	SLC2A5	K08143	molecular_function:fructose transmembrane transporter activity(GO:0005353);molecular_function:glucose transmembrane transporter activity(GO:0005355);cellular_component:plasma membrane(GO:0005886);biological_process:carbohydrate metabolic process(GO:0005975);biological_process:hexose transport(GO:0008645);biological_process:fructose transport(GO:0015755);biological_process:glucose transport(GO:0015758);cellular_component:integral component of membrane(GO:0016021);cellular_component:apical plasma membrane(GO:0016324);molecular_function:substrate-specific transmembrane transporter activity(GO:0022891);biological_process:small molecule metabolic process(GO:0044281);biological_process:transmembrane transport(GO:0055085);biological_process:cellular response to fructose stimulus(GO:0071332)
ENSG00000214559	397	2	0.12	10	0.96	2.899849271	-2.008436072	0.019786881	1 Ups	RP11-323J4.1	_	–
ENSG00000224356	3005	2	0.02	10	0.13	2.899849271	-2.008436072	0.019786881	1 Ups	RP11-151A6.4	_	–
ENSG00000231133	896	2	0.05	10	0.43	2.899849271	-2.008436072	0.019786881	1 Ups	HAR1B	_	–
ENSG00000269302	714	2	0.07	10	0.54	2.899849271	-2.008436072	0.019786881	1 Ups	AC110771.1	_	–
ENSG00000230479	893	39	1.06	5	0.21	-2.270790041	-0.580684241	0.020077659	1 Down	AP000695.6	_	–
ENSG00000178403	2287	0	0	7	0.12	6.201131401	-2.556961829	0.021900161	1 Ups	NEUROG2	K09082	biological_process:neuron migration(GO:0001764);cellular_component:nucleus(GO:0005634);biological_process:transcription, DNA-templated(GO:0006351);biological_process:axon guidance(GO:0007411);biological_process:central nervous system neuron development(GO:0021954);biological_process:forebrain development(GO:0030900);molecular_function:sequence-specific DNA binding(GO:0043565);biological_process:cell fate commitment(GO:0045165);biological_process:positive regulation of neuron differentiation(GO:0045666);biological_process:positive regulation of transcription from RNA polymerase II promoter(GO:0045944);molecular_function:protein dimerization activity(GO:0046983);biological_process:positive regulation of sequence-specific DNA binding transcription factor activity(GO:0051091);molecular_function:E-box binding(GO:0070888)
ENSG00000199575	72	0	0	7	3.71	6.201131401	-2.556961829	0.021900161	1 Ups	SNORD114-1	_	–
ENSG00000201700	73	0	0	7	3.66	6.201131401	-2.556961829	0.021900161	1 Ups	SNORD113-3	_	–

ENSG00000206965	107	0	0	7	2.5	6.201131401	-2.556961829	0.021900161	1	Ups	RNU6-5P	_	_
ENSG00000166546	7165	10	0.03	0	0	-6.047157649	-2.313190369	0.021900161	1	Down	BEAN1	K19324	biological_process:cell death(GO:0008219);cellular_component:integral component of membrane(GO:0016021)
ENSG00000225556	1757	10	0.14	0	0	-6.047157649	-2.313190369	0.021900161	1	Down	C2CD4D	_	_
ENSG00000227258	546	10	0.45	0	0	-6.047157649	-2.313190369	0.021900161	1	Down	SMIM2-AS1	_	_
ENSG00000230880	2317	10	0.11	0	0	-6.047157649	-2.313190369	0.021900161	1	Down	RP11-417J8.3	_	_
ENSG00000234614	506	10	0.48	0	0	-6.047157649	-2.313190369	0.021900161	1	Down	AL450992.2	_	_
ENSG00000251000	418	10	0.58	0	0	-6.047157649	-2.313190369	0.021900161	1	Down	AC008592.3	K00682	_
ENSG00000254701	852	10	0.29	0	0	-6.047157649	-2.313190369	0.021900161	1	Down	RP11-1415C14.4	K01195	_
ENSG00000267344	457	10	0.53	0	0	-6.047157649	-2.313190369	0.021900161	1	Down	CTB-39G8.3	_	_
ENSG00000271524	595	10	0.41	0	0	-6.047157649	-2.313190369	0.021900161	1	Down	CTC-260E6.10	_	_
ENSG00000188818	7267	19	0.06	1	0.01	-3.455908396	-1.558291489	0.022208097	1	Down	ZDHHC11	K18932	molecular_function:zinc ion binding(GO:0008270);cellular_component:integral component of membrane(GO:0016021);molecular_function:transferase activity, transferring acyl groups(GO:0016746)
ENSG00000260645	2123	19	0.22	1	0.02	-3.455908396	-1.558291489	0.022208097	1	Down	RP11-250B2.5	_	_
ENSG00000260388	2468	18	0.18	1	0.02	-3.378548273	-1.616672412	0.022208097	1	Down	LINC00562	_	_
ENSG00000231121	991	5	0.12	17	0.66	2.400846943	-1.285432965	0.023630413	1	Ups	RP1-34H18.1	_	_
ENSG00000203814	2201	6	0.07	17	0.3	2.14499444	-1.236220911	0.023630413	1	Ups	HIST2H2BF	K11253	cellular_component:nucleosome(GO:0000786);molecular_function:DNA binding(GO:0003677);cellular_component:nucleus(GO:0005634);biological_process:nucleosome assembly(GO:0006334);molecular_function:protein heterodimerization activity(GO:0046982)
ENSG00000243055	651	6	0.22	17	1	2.14499444	-1.236220911	0.023630413	1	Ups	GK-AS1	_	_
ENSG00000123358	7290	423	1.41	760	3.98	1.515788857	4.302681189	0.024659558	1	Ups	NR4A1	K04465	molecular_function:RNA polymerase II core promoter proximal region sequence-specific DNA binding transcription factor activity involved in positive regulation of transcription(GO:0001077);biological_process:positive regulation of endothelial cell proliferation(GO:0001938);biological_process:cell migration involved in sprouting angiogenesis(GO:0002042);molecular_function:DNA binding(GO:0003677);molecular_function:steroid hormone receptor activity(GO:0003707);molecular_function:ligand-activated sequence-specific DNA binding RNA polymerase II transcription factor activity(GO:0004879);molecular_function:protein binding(GO:0005515);cellular_component:nucleus(GO:0005634);cellular_component:nucleoplasm(GO:0005654);cellular_component:transcription factor complex(GO:0005667);biological_process:transcription initiation from RNA polymerase II promoter(GO:0006367);;biological_process:signal transduction(GO:0007165);biological_process:epidermal growth factor receptor signaling pathway(GO:0007173);molecular_function:zinc ion binding(GO:0008270);biological_process:fibroblast growth factor receptor signaling pathway(GO:0008543);biological_process:gene expression(GO:0010467);biological_process:intracellular receptor signaling pathway(GO:0030522);biological_process:endothelial cell chemotaxis(GO:0035767);biological_process:skeletal muscle cell differentiation(GO:0035914);biological_process:cellular response to vascular endothelial growth factor stimulus(GO:0035924);biological_process:Fc-epsilon receptor signaling pathway(GO:0038095);molecular_function:protein homodimerization activity(GO:0042803);biological_process:negative regulation of cysteine-type endopeptidase activity involved in apoptotic process(GO:0043154);molecular_function:sequence-specific DNA binding(GO:0043565);biological_process:cellular response to fibroblast growth factor stimulus(GO:0044344);biological_process:innate immune response(GO:0045087);molecular_function:protein heterodimerization activity(GO:0046982);biological_process:neurotrophin TRK receptor signaling pathway(GO:0048011);biological_process:phosphatidylinositol-mediated signaling(GO:0048015);biological_process:cellular response to organic substance(GO:0071310)
ENSG00000237857	1404	1	0.02	9	0.24	3.650014625	-2.20155858	0.027969425	1	Ups	RP11-435O5.2	_	_
ENSG00000271782	279	1	0.09	9	1.23	3.650014625	-2.20155858	0.027969425	1	Ups	RP5-850O15.4	_	_
ENSG00000214353	4853	54	0.27	9	0.07	-1.902892253	-0.105255922	0.028083547	1	Down	VAC14-AS1	_	_
ENSG00000166321	2437	50	0.5	8	0.13	-1.960202248	-0.215484683	0.028732664	1	Down	NUDT13	K03426	cellular_component:mitochondrion(GO:0005739);molecular_function:hydrolase activity(GO:0016787);molecular_function:metal ion binding(GO:0046872)
ENSG00000157654	8203	6	0.02	16	0.07	2.058040211	-1.295749553	0.029323086	1	Ups	PALM2-AKAP2	K16519	biological_process:regulation of cell shape(GO:0008360);cellular_component:membrane(GO:0016020)
ENSG00000205702	2121	6	0.07	16	0.29	2.058040211	-1.295749553	0.029323086	1	Ups	CYP2D7P	K17712	_
ENSG00000221818	7077	6	0.02	16	0.09	2.058040211	-1.295749553	0.029323086	1	Ups	EBF2	K09103	molecular_function:DNA binding(GO:0003677);cellular_component:nucleus(GO:0005634);biological_process:transcription, DNA-templated(GO:0006351);biological_process:regulation of transcription, DNA-templated(GO:0006355);biological_process:multicellular organismal development(GO:0007275);biological_process:positive regulation of transcription, DNA-templated(GO:0045893);molecular_function:metal ion binding(GO:0046872);molecular_function:protein dimerization activity(GO:0046983)
ENSG00000179846	4764	21	0.11	2	0.02	-2.664089749	-1.38700044	0.029721761	1	Down	NKPD1	K08857	cellular_component:integral component of membrane(GO:0016021)
ENSG00000225705	1714	4	0.06	14	0.31	2.433703926	-1.536774383	0.029721761	1	Ups	AC004899.3	K06639	_

ENSG00000100292	2405	11414	115.62	19241	305.7	1.424154754	8.989642462	0.03253895	1	Ups	HMOX1	K00510	biological_process:angiogenesis(GO:0001525);biological_process:response to hypoxia(GO:0001666);biological_process:endothelial cell proliferation(GO:0001935);biological_process:wound healing involved in inflammatory response(GO:0002246);biological_process:negative regulation of leukocyte migration(GO:0002686);molecular_function:heme oxygenase (decyclizing) activity(GO:0004392);molecular_function:phospholipase D activity(GO:0004630);molecular_function:signal transducer activity(GO:0004871);cellular_component:extracellular space(GO:0005615);cellular_component:nucleus(GO:0005634);cellular_component:nucleolus(GO:0005730);cellular_component:endoplasmic reticulum(GO:0005783);cellular_component:endoplasmic reticulum membrane(GO:0005789);cellular_component:cytosol(GO:0005829);cellular_component:caveola(GO:0005901);biological_process:porphyrin-containing compound metabolic process(GO:0006778);biological_process:heme oxidation(GO:0006788);biological_process:cellular iron ion homeostasis(GO:0006879);biological_process:response to oxidative stress(GO:0006979);;biological_process:small GTPase mediated signal transduction(GO:0007264);biological_process:excretion(GO:0007588);biological_process:regulation of blood pressure(GO:0008217);biological_process:cell death(GO:0008219);biological_process:intrinsic apoptotic signaling pathway in response to DNA damage(GO:0008630);biological_process:smooth muscle hyperplasia(GO:0014806);cellular_component:membrane(GO:0016020);molecular_function:enzyme binding(GO:0019899);molecular_function:heme binding(GO:0020037);biological_process:cellular response to nutrient(GO:0031670);biological_process:negative regulation of mast cell cytokine production(GO:0032764);biological_process:erythrocyte homeostasis(GO:0034101);biological_process:low-density lipoprotein particle clearance(GO:0034383);biological_process:response to nicotine(GO:0035094);biological_process:heme catabolic process(GO:0042167);biological_process:response to hydrogen peroxide(GO:0042542);molecular_function:protein homodimerization activity(GO:0042803);biological_process:positive regulation of I-kappaB kinase/NF-kappaB signaling(GO:0043123);biological_process:negative regulation of mast cell degranulation(GO:0043305);biological_process:negative regulation of DNA binding(GO:0043392);biological_process:negative regulation of sequence-specific DNA binding transcription factor activity(GO:0043433);biological_process:negative regulation of neuron apoptotic process(GO:0043524);biological_process:regulation of transcription from RNA polymerase II promoter in response to oxidative stress(GO:0043619);biological_process:response to estrogen(GO:0043627);biological_process:small molecule metabolic process(GO:0044281);biological_process:positive regulation of chemokine biosynthetic process(GO:0045080);biological_process:regulation of angiogenesis(GO:0045765);biological_process:positive regulation of angiogenesis(GO:0045766);biological_process:positive regulation of vasodilation(GO:0045909);molecular_function:metal ion binding(GO:0046872);biological_process:positive regulation of smooth muscle cell proliferation(GO:0048661);biological_process:negative regulation of smooth muscle cell proliferation(GO:0048662);biological_process:regulation of sequence-specific DNA binding transcription factor activity(GO:0051090);biological_process:protein homooligomerization(GO:0051260);biological_process:iron ion homeostasis(GO:0055072);biological_process:transmembrane transport(GO:0055085);biological_process:cellular response to cadmium ion(GO:0071276);biological_process:cellular response to hypoxia(GO:0071456);biological_process:negative regulation of extrinsic apoptotic signaling pathway via death domain receptors(GO:1902042)
ENSG00000170373	872	8	0.22	20	0.88	1.972228742	-0.986740034	0.033540426	1	Ups	CST1	K13897	molecular_function:cysteine-type endopeptidase inhibitor activity(GO:0004869);cellular_component:extracellular region(GO:0005576)
ENSG00000122254	2605	0	0	6	0.09	5.982003394	-2.70169448	0.034782609	1	Ups	HS3ST2	K07808	cellular_component:Golgi membrane(GO:0000139);biological_process:carbohydrate metabolic process(GO:0005975);biological_process:glycosaminoglycan biosynthetic process(GO:0006024);biological_process:circadian rhythm(GO:0007623);molecular_function:sulfotransferase activity(GO:0008146);cellular_component:integral component of membrane(GO:0016021);biological_process:glycosaminoglycan metabolic process(GO:0030203);molecular_function:[heparan sulfate]-glucosamine 3-sulfotransferase 2 activity(GO:0033871);biological_process:small molecule metabolic process(GO:0044281)
ENSG00000180806	1679	0	0	6	0.14	5.982003394	-2.70169448	0.034782609	1	Ups	HOXC9	K09294	molecular_function:sequence-specific DNA binding transcription factor activity(GO:0003700);cellular_component:nucleus(GO:0005634);biological_process:transcription, DNA-templated(GO:0006351);biological_process:anterior/posterior pattern specification(GO:0009952);molecular_function:sequence-specific DNA binding(GO:0043565);biological_process:embryonic skeletal system morphogenesis(GO:0048704)
ENSG00000198019	1392	0	0	6	0.16	5.982003394	-2.70169448	0.034782609	1	Ups	FCGR1B	K06498	biological_process:antigen processing and presentation of peptide antigen via MHC class I(GO:0002474);biological_process:antigen processing and presentation of exogenous peptide antigen via MHC class I, TAP-dependent(GO:0002479);cellular_component:plasma membrane(GO:0005886);biological_process:immune response(GO:0006955);cellular_component:integral component of membrane(GO:0016021);biological_process:cytokine-mediated signaling pathway(GO:0019221);molecular_function:immunoglobulin receptor activity(GO:0019763);molecular_function:IgG binding(GO:0019864);cellular_component:clathrin-coated endocytic vesicle membrane(GO:0030669);cellular_component:early endosome membrane(GO:0031901);biological_process:Fc receptor signaling pathway(GO:0038093);biological_process:antigen processing and presentation of exogenous peptide antigen via MHC class I(GO:0042590);biological_process:interferon-gamma-mediated signaling pathway(GO:0060333)
ENSG00000199934	77	0	0	6	2.98	5.982003394	-2.70169448	0.034782609	1	Ups	SNORD81	_	–
ENSG00000236194	1496	0	0	6	0.15	5.982003394	-2.70169448	0.034782609	1	Ups	AC003104.1	_	–
ENSG00000238258	781	0	0	6	0.29	5.982003394	-2.70169448	0.034782609	1	Ups	RP11-342D11.2	_	–
ENSG00000253414	510	0	0	6	0.45	5.982003394	-2.70169448	0.034782609	1	Ups	RP11-150O12.6	_	–
ENSG00000261431	187	0	0	6	1.23	5.982003394	-2.70169448	0.034782609	1	Ups	RP4-616B8.4	_	–
ENSG00000271522	1517	0	0	6	0.15	5.982003394	-2.70169448	0.034782609	1	Ups	RP11-36B6.1	_	–
ENSG00000109705	2801	9	0.08	0	0	-5.897576652	-2.416157623	0.034782609	1	Down	NKX3-2	K09995	biological_process:skeletal system development(GO:0001501);molecular_function:sequence-specific DNA binding transcription factor activity(GO:0003700);cellular_component:nucleus(GO:0005634);biological_process:transcription from RNA polymerase II promoter(GO:0006366);biological_process:determination of left/right symmetry(GO:0007368);biological_process:pancreas development(GO:0031016);biological_process:negative regulation of chondrocyte differentiation(GO:0032331);biological_process:middle ear morphogenesis(GO:0042474);biological_process:negative regulation of apoptotic process(GO:0043066);molecular_function:sequence-specific DNA binding(GO:0043565);biological_process:spleen development(GO:0048536);biological_process:organ formation(GO:0048645);biological_process:skeletal system morphogenesis(GO:0048705);biological_process:embryonic skeletal system development(GO:0048706);biological_process:intestinal epithelial cell development(GO:0060576)
ENSG00000110934	3976	9	0.06	0	0	-5.897576652	-2.416157623	0.034782609	1	Down	BIN2	K05725	cellular_component:phagocytic cup(GO:0001891);cellular_component:podosome(GO:0002102);molecular_function:protein binding(GO:0005515);molecular_function:phospholipid binding(GO:0005543);cellular_component:cytoplasm(GO:0005737);cellular_component:cell cortex(GO:0005938);biological_process:phagocytosis, engulfment(GO:0006911);cellular_component:cell junction(GO:0030054);cellular_component:cell projection(GO:0042995);biological_process:cell chemotaxis(GO:0060326);biological_process:podosome assembly(GO:0071800);biological_process:membrane tubulation(GO:0097320)

ENSG00000131398	6277	9	0.03	0	0	-5.897576652	-2.416157623	0.034782609	1	Down	KCNC3	K04889	molecular_function:voltage-gated potassium channel activity(GO:0005249);molecular_function:delayed rectifier potassium channel activity(GO:0005251);cellular_component:plasma membrane(GO:0005886);biological_process:potassium ion transport(GO:0006813);biological_process:synaptic transmission(GO:0007268);cellular_component:voltage-gated potassium channel complex(GO:0008076);biological_process:cell death(GO:0008219);cellular_component:axolemma(GO:0030673);cellular_component:neuromuscular junction(GO:0031594);cellular_component:dendrite membrane(GO:0032590);cellular_component:neuronal cell body membrane(GO:0032809);cellular_component:axon terminus(GO:0043679);biological_process:regulation of neurotransmitter secretion(GO:0046928);biological_process:protein homooligomerization(GO:0051260)
ENSG00000158813	6401	9	0.03	0	0	-5.897576652	-2.416157623	0.034782609	1	Down	EDA	K05480	molecular_function:receptor binding(GO:0005102);molecular_function:tumor necrosis factor receptor binding(GO:0005164);molecular_function:protein binding(GO:0005515);cellular_component:collagen trimer(GO:0005581);cellular_component:endoplasmic reticulum membrane(GO:0005789);cellular_component:cytoskeleton(GO:0005856);cellular_component:plasma membrane(GO:0005886);cellular_component:integral component of plasma membrane(GO:0005887);biological_process:immune response(GO:0006955);biological_process:cell-matrix adhesion(GO:0007160);biological_process:signal transduction(GO:0007165);biological_process:ectoderm development(GO:0007398);biological_process:gene expression(GO:0010467);cellular_component:membrane(GO:0016020);cellular_component:integral component of membrane(GO:0016021);biological_process:cell differentiation(GO:0030154);biological_process:positive regulation of NF-kappaB import into nucleus(GO:0042346);biological_process:odontogenesis of dentin-containing tooth(GO:0042475);biological_process:positive regulation of I-kappaB kinase/NF-kappaB signaling(GO:0043123);biological_process:pigmentation(GO:0043473);cellular_component:apical part of cell(GO:0045177);biological_process:positive regulation of NF-kappaB transcription factor activity(GO:0051092);biological_process:salivary gland cavitation(GO:0060662);biological_process:hair follicle placode formation(GO:0060789);biological_process:trachea gland development(GO:0061153);biological_process:positive regulation of canonical Wnt signaling pathway(GO:0090263)
ENSG00000207234	107	9	2.05	0	0	-5.897576652	-2.416157623	0.034782609	1	Down	RNU6-125P	_	–
ENSG00000228013	1756	9	0.12	0	0	-5.897576652	-2.416157623	0.034782609	1	Down	RP11-350G8.5	_	–
ENSG00000234936	398	9	0.55	0	0	-5.897576652	-2.416157623	0.034782609	1	Down	AC010883.5	_	–
ENSG00000237021	2081	9	0.11	0	0	-5.897576652	-2.416157623	0.034782609	1	Down	RP3-486I3.7	_	–
ENSG00000253392	776	9	0.28	0	0	-5.897576652	-2.416157623	0.034782609	1	Down	AC006277.2	_	–
ENSG00000259584	864	9	0.25	0	0	-5.897576652	-2.416157623	0.034782609	1	Down	RP11-521C20.2	_	–
ENSG00000266803	689	9	0.32	0	0	-5.897576652	-2.416157623	0.034782609	1	Down	RP1-77H15.1	_	–
ENSG00000267069	733	9	0.3	0	0	-5.897576652	-2.416157623	0.034782609	1	Down	RP11-64C12.8	_	–
ENSG00000109132	3090	8	0.06	0	0	-5.730673565	-2.527541636	0.034782609	1	Down	PHOX2B	K09330	cellular_component:nuclear chromatin(GO:0000790);molecular_function:RNA polymerase II regulatory region sequence-specific DNA binding(GO:0000977);molecular_function:RNA polymerase II core promoter proximal region sequence-specific DNA binding(GO:0000978);biological_process:neuron migration(GO:0001764);biological_process:regulation of respiratory gaseous exchange by neurological system process(GO:0002087);biological_process:noradrenergic neuron differentiation(GO:0003357);biological_process:brainstem development(GO:0003360);molecular_function:sequence-specific DNA binding transcription factor activity(GO:0003700);cellular_component:nucleus(GO:0005634);biological_process:transcription, DNA-templated(GO:0006351);biological_process:negative regulation of cell proliferation(GO:0008285);biological_process:glial cell differentiation(GO:0010001);biological_process:cell differentiation in hindbrain(GO:0021533);biological_process:medullary reticular formation development(GO:0021723);biological_process:hindbrain tangential cell migration(GO:0021934);biological_process:skeletal muscle cell differentiation(GO:0035914);molecular_function:sequence-specific DNA binding(GO:0043565);biological_process:negative regulation of neuron differentiation(GO:0045665);biological_process:positive regulation of neuron differentiation(GO:0045666);biological_process:positive regulation of transcription from RNA polymerase II promoter(GO:0045944);biological_process:autonomic nervous system development(GO:0048483);biological_process:enteric nervous system development(GO:0048484);biological_process:sympathetic nervous system development(GO:0048485);biological_process:parasympathetic nervous system development(GO:0048486);biological_process:inner ear development(GO:0048839);biological_process:efferent axon development in a lateral line nerve(GO:0048894);biological_process:respiratory system development(GO:0060541);biological_process:retrotrapezoid nucleus neuron differentiation(GO:0061452);biological_process:dopaminergic neuron differentiation(GO:0071542);biological_process:cellular response to BMP stimulus(GO:0071773);biological_process:neural crest cell migration involved in autonomic nervous system development(GO:1901166)
ENSG00000135127	4793	8	0.04	0	0	-5.730673565	-2.527541636	0.034782609	1	Down	CCDC64	K16756	cellular_component:cytoplasm(GO:0005737);cellular_component:centrosome(GO:0005813);molecular_function:Rab GTPase binding(GO:0017137);biological_process:neuron projection development(GO:0031175);molecular_function:dynactin binding(GO:0034452);biological_process:Golgi to secretory granule transport(GO:0055107)
ENSG00000148600	6189	8	0.03	0	0	-5.730673565	-2.527541636	0.034782609	1	Down	CDHR1	K16501	molecular_function:calcium ion binding(GO:0005509);cellular_component:plasma membrane(GO:0005886);cellular_component:integral component of plasma membrane(GO:0005887);biological_process:homophilic cell adhesion via plasma membrane adhesion molecules(GO:0007156);cellular_component:integral component of membrane(GO:0016021);cellular_component:photoreceptor outer segment membrane(GO:0042622);biological_process:photoreceptor cell maintenance(GO:0045494)
ENSG00000197153	842	8	0.23	0	0	-5.730673565	-2.527541636	0.034782609	1	Down	HIST1H3J	K11253	–
ENSG00000212743	2446	8	0.08	0	0	-5.730673565	-2.527541636	0.034782609	1	Down	DKFZP667F0711	_	–
ENSG00000222788	79	8	2.47	0	0	-5.730673565	-2.527541636	0.034782609	1	Down	RNU2-38P	_	–
ENSG00000223309	104	8	1.87	0	0	-5.730673565	-2.527541636	0.034782609	1	Down	RNU6-722P	_	–
ENSG00000224712	1473	8	0.13	0	0	-5.730673565	-2.527541636	0.034782609	1	Down	NPIPA3	K01896	–
ENSG00000225905	543	8	0.36	0	0	-5.730673565	-2.527541636	0.034782609	1	Down	RP4-758J18.7	_	–
ENSG00000228044	2201	8	0.09	0	0	-5.730673565	-2.527541636	0.034782609	1	Down	RP4-781K5.4	_	–
ENSG00000228251	529	8	0.37	0	0	-5.730673565	-2.527541636	0.034782609	1	Down	AC012442.6	_	–
ENSG00000229400	837	8	0.23	0	0	-5.730673565	-2.527541636	0.034782609	1	Down	RP11-3L21.2	_	–
ENSG00000233967	935	8	0.21	0	0	-5.730673565	-2.527541636	0.034782609	1	Down	RP11-250B2.3	_	–

ENSG00000236699	7579	8	0.03	0	0	-5.730673565	-2.527541636	0.034782609	1	Down	ARHGEF38	K13183	molecular_function:Rho guanyl-nucleotide exchange factor activity(GO:0005089);biological_process:regulation of Rho protein signal transduction(GO:0035023)
ENSG00000239219	7977	8	0.02	0	0	-5.730673565	-2.527541636	0.034782609	1	Down	RP11-379K17.4	_	–
ENSG00000254740	894	8	0.22	0	0	-5.730673565	-2.527541636	0.034782609	1	Down	RP11-334E6.3	_	–
ENSG00000267715	412	8	0.47	0	0	-5.730673565	-2.527541636	0.034782609	1	Down	CTB-25B13.6	_	–
ENSG00000269794	1213	8	0.16	0	0	-5.730673565	-2.527541636	0.034782609	1	Down	AC010642.2	K09228	–
ENSG00000171051	2594	7	0.07	18	0.27	2.009763837	-1.133272095	0.034945041	1	Ups	FPR1	K04172	biological_process:activation of MAPK activity(GO:0000187);molecular_function:receptor activity(GO:0004872);molecular_function:N-formyl peptide receptor activity(GO:0004982);molecular_function:protein binding(GO:0005515);cellular_component:endosome(GO:0005768);cellular_component:plasma membrane(GO:0005886);biological_process:movement of cell or subcellular component(GO:0006928);biological_process:chemotaxis(GO:0006935);biological_process:signal transduction(GO:0007165);biological_process:G-protein coupled receptor signaling pathway(GO:0007186);biological_process:adenylate cyclase-modulating G-protein coupled receptor signaling pathway(GO:0007188);biological_process:nitric oxide mediated signal transduction(GO:0007263);cellular_component:integral component of membrane(GO:0016021)
ENSG00000207971	88	7	1.94	18	7.82	2.009763837	-1.133272095	0.034945041	1	Ups	MIR125B1	_	–
ENSG00000198417	1889	11	0.14	24	0.49	1.78210579	-0.697894379	0.036252813	1	Ups	MT1F	K14739	molecular_function:copper ion binding(GO:0005507);cellular_component:nucleus(GO:0005634);cellular_component:cytoplasm(GO:0005737);molecular_function:zinc ion binding(GO:0008270);biological_process:negative regulation of growth(GO:0045926);molecular_function:cadmium ion binding(GO:0046870);molecular_function:metal ion binding(GO:0046872);cellular_component:perinuclear region of cytoplasm(GO:0048471);biological_process:cellular response to cadmium ion(GO:0071276);biological_process:cellular response to zinc ion(GO:0071294)
ENSG00000267219	585	24	1	3	0.2	-2.292759112	-1.18917389	0.036520717	1	Down	AC010504.2	_	–
ENSG00000196337	3387	20	0.14	2	0.02	-2.594223931	-1.438534111	0.038199172	1	Down	CGB7	K10045	molecular_function:hormone activity(GO:0005179);cellular_component:extracellular region(GO:0005576)
ENSG00000128016	2010	983	11.91	1613	30.66	1.385131664	5.423837505	0.038250559	1	Ups	ZFP36	K15308	biological_process:negative regulation of transcription from RNA polymerase II promoter(GO:0000122);biological_process:nuclear-transcribed mRNA catabolic process, deadenylation-dependent decay(GO:0000288);molecular_function:DNA binding(GO:0003677);molecular_function:single-stranded RNA binding(GO:0003727);molecular_function:mRNA binding(GO:0003729);molecular_function:protein binding(GO:0005515);cellular_component:nucleus(GO:0005634);cellular_component:cytoplasm(GO:0005737);cellular_component:cytosol(GO:0005829);biological_process:mRNA catabolic process(GO:0006402);biological_process:response to stress(GO:0006950);biological_process:gene expression(GO:0010467);cellular_component:cytoplasmic stress granule(GO:0010494);biological_process:RNA metabolic process(GO:0016070);biological_process:mRNA metabolic process(GO:0016071);molecular_function:AU-rich element binding(GO:0017091);molecular_function:protein kinase binding(GO:0019901);molecular_function:C-C chemokine binding(GO:0019957);biological_process:regulation of tumor necrosis factor production(GO:0032680);biological_process:negative regulation of translation involved in gene silencing by miRNA(GO:0035278);molecular_function:metal ion binding(GO:0046872);biological_process:positive regulation of nuclear-transcribed mRNA poly(A) tail shortening(GO:0060213);biological_process:3'-UTR-mediated mRNA stabilization(GO:0070935);molecular_function:14-3-3 protein binding(GO:0071889);biological_process:positive regulation of nuclear-transcribed mRNA catabolic process, deadenylation-dependent decay(GO:1900153)
ENSG00000257242	3638	15	0.1	32	0.34	1.753545933	-0.297789324	0.039450496	1	Ups	C12orf79	_	–
ENSG00000162654	6127	3	0.01	11	0.07	2.485838739	-1.838067336	0.039696281	1	Ups	GBP4	K17985	molecular_function:GTPase activity(GO:0003924);molecular_function:GTP binding(GO:0005525);cellular_component:nucleus(GO:0005634);cellular_component:cytoplasm(GO:0005737)
ENSG00000259518	2568	66	0.63	13	0.19	-1.665854795	0.203752494	0.039747057	1	Down	RP11-597K23.2	_	–
ENSG00000129244	3276	1	0.01	8	0.09	3.48200053	-2.315161357	0.039970491	1	Ups	ATP1B2	K01540	molecular_function:sodium:potassium-exchanging ATPase activity(GO:0005391);molecular_function:protein binding(GO:0005515);cellular_component:cytoplasm(GO:0005737);cellular_component:plasma membrane(GO:0005886);cellular_component:sodium:potassium-exchanging ATPase complex(GO:0005890);biological_process:transport(GO:0006810);biological_process:potassium ion transport(GO:0006813);biological_process:sodium ion transport(GO:0006814);biological_process:cell adhesion(GO:0007155);biological_process:blood coagulation(GO:0007596);biological_process:ion transmembrane transport(GO:0034220);biological_process:leukocyte migration(GO:0050900);biological_process:transmembrane transport(GO:0055085)
ENSG00000134640	1956	1	0.01	8	0.16	3.48200053	-2.315161357	0.039970491	1	Ups	MTNR1B	K04286	molecular_function:G-protein coupled receptor activity(GO:0004930);cellular_component:plasma membrane(GO:0005886);cellular_component:integral component of plasma membrane(GO:0005887);biological_process:G-protein coupled receptor signaling pathway, coupled to cyclic nucleotide second messenger(GO:0007187);biological_process:synaptic transmission(GO:0007268);molecular_function:melatonin receptor activity(GO:0008502);cellular_component:integral component of membrane(GO:0016021);biological_process:glucose homeostasis(GO:0042593);biological_process:regulation of insulin secretion(GO:0050796)
ENSG00000135625	2376	1	0.01	8	0.13	3.48200053	-2.315161357	0.039970491	1	Ups	EGR4	K12498	molecular_function:DNA binding(GO:0003677);molecular_function:sequence-specific DNA binding transcription factor activity(GO:0003700);cellular_component:nucleus(GO:0005634);biological_process:transcription, DNA-templated(GO:0006351);biological_process:positive regulation of cell proliferation(GO:0008284);molecular_function:metal ion binding(GO:0046872)
ENSG00000196796	1350	1	0.02	8	0.23	3.48200053	-2.315161357	0.039970491	1	Ups	CTB-134H23.2	_	–
ENSG00000268754	465	1	0.05	8	0.66	3.48200053	-2.315161357	0.039970491	1	Ups	LINC01081	_	–
ENSG00000271734	470	1	0.05	8	0.65	3.48200053	-2.315161357	0.039970491	1	Ups	RP1-111B22.3	_	–
ENSG00000084628	2987	2	0.02	8	0.1	2.581362633	-2.214173088	0.039970491	1	Ups	NKAIN1	_	cellular_component:plasma membrane(GO:0005886);cellular_component:integral component of membrane(GO:0016021)

ENSG00000167588	3991	2	0.01	8	0.08	2.581362633	-2.214173088	0.039970491	1	Ups	GPD1	K00006	molecular_function:glycerol-3-phosphate dehydrogenase [NAD+] activity(GO:0004367);molecular_function:glycerol-3-phosphate dehydrogenase activity(GO:0004368);cellular_component:cytoplasm(GO:0005737);cellular_component:mitochondrion(GO:0005739);cellular_component:cytosol(GO:0005829);biological_process:gluconeogenesis(GO:0006094);biological_process:NADH oxidation(GO:0006116);biological_process:phospholipid metabolic process(GO:0006644);biological_process:phosphatidic acid biosynthetic process(GO:0006654);cellular_component:glycerol-3-phosphate dehydrogenase complex(GO:0009331);biological_process:triglyceride biosynthetic process(GO:0019432);molecular_function:protein homodimerization activity(GO:0042803);biological_process:cellular lipid metabolic process(GO:0044255);biological_process:small molecule metabolic process(GO:0044281);biological_process:glycerol-3-phosphate catabolic process(GO:0046168);biological_process:glycerophospholipid biosynthetic process(GO:0046474);molecular_function:NAD binding(GO:0051287);biological_process:cellular response to cAMP(GO:0071320);biological_process:cellular response to tumor necrosis factor(GO:0071356)
ENSG00000223486	2691	2	0.02	8	0.11	2.581362633	-2.214173088	0.039970491	1	Ups	AC092198.1	_	–
ENSG00000271992	466	2	0.1	8	0.66	2.581362633	-2.214173088	0.039970491	1	Ups	RP11-42O15.3	_	–
ENSG00000248966	2752	26	0.23	48	0.67	1.549711479	0.323294543	0.042332043	1	Ups	CTD-2089O24.1	K13087	–
ENSG00000260160	675	26	0.94	4	0.23	-2.003771926	-1.05465585	0.042605988	1	Down	CTC-471J1.2	_	–
ENSG00000230499	412	5	0.3	15	1.39	2.22135952	-1.411234866	0.045651874	1	Ups	AC108463.1	_	–
ENSG00000091129	7939	6	0.02	15	0.07	1.965507017	-1.357571915	0.045651874	1	Ups	NRCAM	K06756	biological_process:angiogenesis(GO:0001525);biological_process:neuron migration(GO:0001764);cellular_component:plasma membrane(GO:0005886);cellular_component:integral component of plasma membrane(GO:0005887);biological_process:axonogenesis(GO:0007409);biological_process:axon guidance(GO:0007411);biological_process:axonal fasciculation(GO:0007413);biological_process:synapse assembly(GO:0007416);biological_process:central nervous system development(GO:0007417);biological_process:protein localization(GO:0008104);cellular_component:external side of plasma membrane(GO:0009897);biological_process:regulation of neuron projection development(GO:0010975);biological_process:single organismal cell-cell adhesion(GO:0016337);biological_process:neuronal action potential propagation(GO:0019227);cellular_component:axon(GO:0030424);molecular_function:ankyrin binding(GO:0030506);biological_process:regulation of axon extension(GO:0030516);biological_process:retinal ganglion cell axon guidance(GO:0031290);cellular_component:neuron projection(GO:0043005);cellular_component:axon initial segment(GO:0043194);biological_process:clustering of voltage-gated sodium channels(GO:0045162);cellular_component:synapse(GO:0045202);biological_process:positive regulation of neuron differentiation(GO:0045666);molecular_function:protein binding involved in heterotypic cell-cell adhesion(GO:0086080)
ENSG00000223612	704	151	5.23	244	13.24	1.36222411	2.705760018	0.046327477	1	Ups	RP11-289I10.2	_	–
ENSG00000006128	1888	8	0.1	19	0.38	1.898592547	-1.037193026	0.048036813	1	Ups	TAC1	K05239	biological_process:positive regulation of acute inflammatory response(GO:0002675);molecular_function:protein binding(GO:0005515);cellular_component:extracellular region(GO:0005576);cellular_component:extracellular space(GO:0005615);cellular_component:plasma membrane(GO:0005886);biological_process:inflammatory response(GO:0006954);biological_process:positive regulation of cytosolic calcium ion concentration(GO:0007204);biological_process:tachykinin receptor signaling pathway(GO:0007217);biological_process:neuropeptide signaling pathway(GO:0007218);biological_process:cell-cell signaling(GO:0007267);biological_process:synaptic transmission(GO:0007268);biological_process:insemination(GO:0007320);biological_process:long-term memory(GO:0007616);biological_process:regulation of blood pressure(GO:0008217);biological_process:associative learning(GO:0008306);biological_process:detection of abiotic stimulus(GO:0009582);biological_process:response to hormone(GO:0009725);biological_process:negative regulation of heart rate(GO:0010459);biological_process:positive regulation of epithelial cell migration(GO:0010634);biological_process:sensory perception of pain(GO:0019233);cellular_component:axon(GO:0030424);molecular_function:substance P receptor binding(GO:0031835);biological_process:positive regulation of synaptic transmission, cholinergic(GO:0032224);biological_process:positive regulation of synaptic transmission, GABAergic(GO:0032230);biological_process:response to lipopolysaccharide(GO:0032496);biological_process:positive regulation of renal sodium excretion(GO:0035815);cellular_component:neuronal cell body(GO:0043025);biological_process:response to morphine(GO:0043278);biological_process:positive regulation of action potential(GO:0045760);biological_process:positive regulation of saliva secretion(GO:0046878);biological_process:response to pain(GO:0048265);biological_process:positive regulation of lymphocyte proliferation(GO:0050671);biological_process:positive regulation of stress fiber assembly(GO:0051496);biological_process:positive regulation of corticosterone secretion(GO:2000854)
ENSG00000147127	1344	8	0.15	19	0.54	1.898592547	-1.037193026	0.048036813	1	Ups	RAB41	K07896	molecular_function:GTP binding(GO:0005525);biological_process:small GTPase mediated signal transduction(GO:0007264);biological_process:protein transport(GO:0015031)
ENSG00000185689	3322	19	0.14	2	0.02	-2.520801744	-1.49211998	0.049335813	1	Down	C6orf201	K13239	–
ENSG00000232926	682	18	0.64	2	0.11	-2.443441621	-1.547928479	0.049335813	1	Down	AC000078.5	_	–
ENSG00000269923	2835	18	0.15	2	0.03	-2.443441621	-1.547928479	0.049335813	1	Down	RP11-214K3.24	_	–
ENSG00000272275	393	18	1.12	2	0.19	-2.443441621	-1.547928479	0.049335813	1	Down	RP11-791G15.2	_	–
ENSG00000084710	6828	48	0.17	9	0.05	-1.733478489	-0.230430444	0.049705669	1	Down	EFR3B	K04257	–
