# Supplementary material for: Ozone ultrafine bubble water induces the cellular signaling involved in oxidative stress responses in human periodontal ligament fibroblasts
Source: Sci Technol Adv Mater. 2019 Jun 13;20(1):589–98. doi: 10.1080/14686996.2019.1614980 (PMC6586087; doi:10.1080/14686996.2019.1614980)
Supplement: Supplemental Material [file TSTA_A_1614980_SM5109.zip › suppl2.pdf]

Table S2

Pathway ID	Pathway	Category	DEGs with pathway annotation (58)	All genes with pathway annotation (16833)	Pvalue	Qvalue	Genelist	KOlist	Pathwaylink
ko04380	Osteoclast differentiation	Organismal Systems	3(5.17%)	184(1.09%)	3.70E-03	3.33E-02	ENSG00000170345,ENSG00000125740,ENSG00000198019,	K04379,K09029,K06498,	http://www.kegg.jp/kegg-bin/show_pathway?scale=1.0&query=&map=ko04380&use=reference&multi_query=K04379+red%0D%0AK09029+red%0D%0AK06498+red%0D%0A
ko04010	MAPK signaling pathway	Environmental Information Processing	5(8.62%)	411(2.44%)	2.83E-03	2.99E-02	ENSG00000170345,ENSG00000173110,ENSG00000225217,ENSG00000156427,ENSG0	K04379,K03283,K04358,K04465,	http://www.kegg.jp/kegg-bin/show_pathway?scale=1.0&query=&map=ko04010&use=reference&multi_query=K04379+red%0D%0AK03283+red%0D%0AK04358+red%0D%0AK04465+red%0D%0A
ko05034	Alcoholism	Human Diseases	6(10.34%)	308(1.83%)	8.67E-05	3.12E-03	ENSG00000197846,ENSG00000125740,ENSG00000203814,ENSG00000203814,ENSG0	K11252,K09029,K11253,	http://www.kegg.jp/kegg-bin/show_pathway?scale=1.0&query=&map=ko05034&use=reference&multi_query=K11252+red%0D%0AK09029+red%0D%0AK11253+green%0D%0A
ko00944	Flavone and flavonol biosynthesis	Metabolism	1(1.72%)	20(0.12%)	2.13E-03	2.56E-02	ENSG00000254701,	K01195,	http://www.kegg.jp/kegg-bin/show_pathway?scale=1.0&query=&map=ko00944&use=reference&multi_query=K01195+blue%0D%0A
ko01522	Endocrine resistance		3(5.17%)	172(1.02%)	2.91E-03	2.99E-02	ENSG00000170345,ENSG00000205702,ENSG00000110934,	K04379,K17712,K05725,	http://www.kegg.jp/kegg-bin/show_pathway?scale=1.0&query=&map=ko01522&use=reference&multi_query=K04379+red%0D%0AK17712+red%0D%0AK05725+blue%0D%0A
ko04973	Carbohydrate digestion and absorption	Organismal Systems	2(3.45%)	56(0.33%)	9.45E-04	1.70E-02	ENSG00000142583,ENSG00000129244,	K08143,K01540,	http://www.kegg.jp/kegg-bin/show_pathway?scale=1.0&query=&map=ko04973&use=reference&multi_query=K08143+red%0D%0AK01540+red%0D%0A
ko04213	Longevity regulating pathway - multiple species		2(3.45%)	103(0.61%)	5.38E-03	3.69E-02	ENSG00000173110,ENSG00000225217,	K03283,	http://www.kegg.jp/kegg-bin/show_pathway?scale=1.0&query=&map=ko04213&use=reference&multi_query=K03283+red%0D%0A
ko04978	Mineral absorption	Organismal Systems	8(13.79%)	107(0.64%)	9.89E-11	1.42E-08	ENSG00000125144,ENSG00000187193,ENSG00000170385,ENSG00000205364,ENSG0	K14739,K14688,K00510,K01540,	http://www.kegg.jp/kegg-bin/show_pathway?scale=1.0&query=&map=ko04978&use=reference&multi_query=K14739+red%0D%0AK14688+red%0D%0AK00510+red%0D%0AK01540+red%0D%0A
ko04212	Longevity regulating pathway - worm		5(8.62%)	183(1.09%)	3.85E-05	1.85E-03	ENSG00000125144,ENSG00000187193,ENSG00000205364,ENSG00000169715,ENSG0	K14739,	http://www.kegg.jp/kegg-bin/show_pathway?scale=1.0&query=&map=ko04212&use=reference&multi_query=K14739+red%0D%0A
ko00534	Glycosaminoglycan biosynthesis - heparan sulfate / heparin	Metabolism	1(1.72%)	29(0.17%)	4.46E-03	3.57E-02	ENSG00000122254,	K07808,	http://www.kegg.jp/kegg-bin/show_pathway?scale=1.0&query=&map=ko00534&use=reference&multi_query=K07808+red%0D%0A
ko04080	Neuroactive ligand-receptor interaction	Environmental Information Processing	5(8.62%)	443(2.63%)	4.09E-03	3.47E-02	ENSG00000175591,ENSG00000236699,ENSG00000171051,ENSG00000134640,ENSG0	K04269,K04135,K04172,K04286,K05239,	http://www.kegg.jp/kegg-bin/show_pathway?scale=1.0&query=&map=ko04080&use=reference&multi_query=K04269+red%0D%0AK04135+blue%0D%0AK04172+red%0D%0AK04286+red%0D%0AK0523

ko04950	Maturity onset diabetes of the young	Human Diseases	1(1.72%)	31(0.18%)	5.09E-03	3.66E-02	ENSG00000114315,	K06054,	http://www.kegg.jp/kegg-bin/show_pathway?scale=1.0&query=&map=ko04950&use=reference&multi_query=K06054+red%0D%0A
ko04370	VEGF signaling pathway	Environmental Information Processing	2(3.45%)	99(0.59%)	4.82E-03	3.65E-02	ENSG00000073756,ENSG00000110934,	K11987,K05725,	http://www.kegg.jp/kegg-bin/show_pathway?scale=1.0&query=&map=ko04370&use=reference&multi_query=K11987+red%0D%0AK05725+blue%0D%0A
ko00860	Porphyrin and chlorophyll metabolism	Metabolism	2(3.45%)	106(0.63%)	5.83E-03	3.81E-02	ENSG00000254701,ENSG00000100292,	K01195,K00510,	http://www.kegg.jp/kegg-bin/show_pathway?scale=1.0&query=&map=ko00860&use=reference&multi_query=K01195+blue%0D%0AK00510+red%0D%0A
ko04657	IL-17 signaling pathway		3(5.17%)	137(0.81%)	1.27E-03	2.03E-02	ENSG00000170345,ENSG00000125740,ENSG00000073756,	K04379,K09029,K11987,	http://www.kegg.jp/kegg-bin/show_pathway?scale=1.0&query=&map=ko04657&use=reference&multi_query=K04379+red%0D%0AK09029+red%0D%0AK11987+red%0D%0A
ko05322	Systemic lupus erythematosus	Human Diseases	7(12.07%)	355(2.11%)	2.76E-05	1.85E-03	ENSG00000197846,ENSG00000077522,ENSG00000203814,ENSG00000203814,ENSG0	K11252,K05699,K11253,K06498,	http://www.kegg.jp/kegg-bin/show_pathway?scale=1.0&query=&map=ko05322&use=reference&multi_query=K11252+red%0D%0AK05699+red%0D%0AK11253+green%0D%0AK06498+red%0D%0A
ko04970	Salivary secretion	Organismal Systems	3(5.17%)	149(0.89%)	1.73E-03	2.49E-02	ENSG00000170373,ENSG00000236699,ENSG00000129244,	K13897,K04135,K01540,	http://www.kegg.jp/kegg-bin/show_pathway?scale=1.0&query=&map=ko04970&use=reference&multi_query=K13897+red%0D%0AK04135+blue%0D%0AK01540+red%0D%0A
ko05224	Breast cancer		4(6.90%)	206(1.22%)	7.08E-04	1.46E-02	ENSG00000114315,ENSG00000170345,ENSG00000163909,ENSG00000156427,	K06054,K04379,K09091,K04358,	http://www.kegg.jp/kegg-bin/show_pathway?scale=1.0&query=&map=ko05224&use=reference&multi_query=K06054+red%0D%0AK04379+red%0D%0AK09091+red%0D%0AK04358+red%0D%0A
ko05031	Amphetamine addiction	Human Diseases	3(5.17%)	86(0.51%)	2.18E-04	6.29E-03	ENSG00000170345,ENSG00000125740,ENSG00000198576,	K04379,K09029,K15867,	http://www.kegg.jp/kegg-bin/show_pathway?scale=1.0&query=&map=ko05031&use=reference&multi_query=K04379+red%0D%0AK09029+red%0D%0AK15867+red%0D%0A
ko03460	Fanconi anemia pathway	Genetic Information Processing	2(3.45%)	112(0.67%)	6.78E-03	4.25E-02	ENSG00000114315,ENSG00000174384,	K06054,K10858,	http://www.kegg.jp/kegg-bin/show_pathway?scale=1.0&query=&map=ko03460&use=reference&multi_query=K06054+red%0D%0AK10858+blue%0D%0A
ko04726	Serotonergic synapse	Organismal Systems	3(5.17%)	153(0.91%)	1.90E-03	2.49E-02	ENSG00000073756,ENSG00000205702,ENSG00000205702,	K11987,K17712,	http://www.kegg.jp/kegg-bin/show_pathway?scale=1.0&query=&map=ko04726&use=reference&multi_query=K11987+red%0D%0AK17712+red%0D%0A
ko04915	Estrogen signaling pathway	Organismal Systems	3(5.17%)	178(1.06%)	3.29E-03	3.16E-02	ENSG00000170345,ENSG00000173110,ENSG00000225217,	K04379,K03283,	http://www.kegg.jp/kegg-bin/show_pathway?scale=1.0&query=&map=ko04915&use=reference&multi_query=K04379+red%0D%0AK03283+red%0D%0A
ko05140	Leishmaniasis	Human Diseases	4(6.90%)	206(1.22%)	7.08E-04	1.46E-02	ENSG00000170345,ENSG00000073756,ENSG00000111679,ENSG00000198019,	K04379,K11987,K05697,K06498,	http://www.kegg.jp/kegg-bin/show_pathway?scale=1.0&query=&map=ko05140&use=reference&multi_query=K04379+red%0D%0AK11987+red%0D%0AK05697+red%0D%0AK06498+red%0D%0A
